# Optical Constants of Several Multilayer Transition Metal Dichalcogenides Measured by Spectroscopic Ellipsometry in the 300–1700 nm Range: High Index, Anisotropy, and Hyperbolicity

**DOI:** 10.1021/acsphotonics.2c00433

**Published:** 2022-06-07

**Authors:** Battulga Munkhbat, Piotr Wróbel, Tomasz J. Antosiewicz, Timur O. Shegai

**Affiliations:** † Department of Physics, Chalmers University of Technology, SE-412 96 Gothenburg, Sweden; ‡ Department of Photonics Engineering, Technical University of Denmark, 2800 Kongens Lyngby, Denmark; ¶ Faculty of Physics, University of Warsaw, Pasteura 5, PL-02-093 Warsaw, Poland

**Keywords:** semiconductors, transition metal dichalcogenides, anisotropy, high-index, nanophotonics

## Abstract

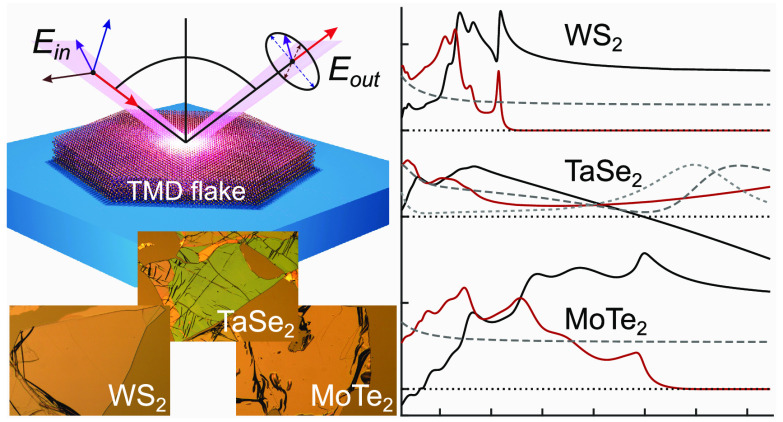

Transition metal dichalcogenides (TMDs) attract significant attention due to their remarkable optical and excitonic properties. It was understood already in the 1960s and recently rediscovered that many TMDs possess a high refractive index and optical anisotropy, which make them attractive for nanophotonic applications. However, accurate analysis and predictions of nanooptical phenomena require knowledge of dielectric constants along both in- and out-of-plane directions and over a broad spectral range, information that is often inaccessible or incomplete. Here, we present an experimental study of optical constants from several exfoliated TMD multilayers obtained using spectroscopic ellipsometry in the broad range of 300–1700 nm. The specific materials studied include semiconducting WS_2_, WSe_2_, MoS_2_, MoSe_2_, and MoTe_2_, as well as in-plane anisotropic ReS_2_ and WTe_2_ and metallic TaS_2_, TaSe_2_, and NbSe_2_. The extracted parameters demonstrate a high index (*n* up to ∼4.84 for MoTe_2_), significant anisotropy (*n*_∥_ – *n*_⊥_ ≈ 1.54 for MoTe_2_), and low absorption in the near-infrared region. Moreover, metallic TMDs show potential for combined plasmonic–dielectric behavior and hyperbolicity, as their plasma frequency occurs at around ∼1000–1300 nm depending on the material. The knowledge of optical constants of these materials opens new experimental and computational possibilities for further development of all-TMD nanophotonics.

## Introduction

One of the main reasons for the growing interest in 2D semiconductors stems from the recent discovery of a direct band gap in monolayer MoS_2_.^1,2^ In addition to exciting exciton physics in transition metal dichalcogenide (TMD) monolayers,^3^ multilayer TMDs possess a number of attractive optical, electronic, and mechanical properties. The prospects of multilayer TMDs for optics and photonics have been discussed already in the 1960s.^4^ For example, due to their van der Waals (vdW) nature, TMDs are naturally anisotropic, which is reflected in their physical and optical properties.^5−9^ Due to large oscillator strengths of electronic excitations around the A-, B- and C-exciton bands, which are stable in both mono- and multilayers even at room temperature, TMD materials possess high refractive indices in the visible and near-infrared range.^10−13^ Moreover, below the A-exciton absorption band, there is a relatively broad region of low loss.^7,11^

These observations have led to a recently renewed interest in TMD optics and nanophotonics. This interest has grown even more after publication of several nanopatterning methods of TMDs and studies of optical phenomena in resulting TMD nanostructures.^14,15^ Recent observations include high-index Mie resonances and anapole states in WS_2_ nanodisks,^14^ optical anisotropy in TMD slabs^7,9^ and nanocones,^16^ self-hybridization in TMD slabs^17,18^ and nanotubes,^19^ optical modes in lattices of TMD nanostructures,^20^ improved second-harmonic generation in WS_2_ and MoS_2_ nanodisks,^21,22^ high-index metamaterials,^23^ nanoholes down to ∼20 nm,^24^ dimer nanoantennas,^25^ and TMD metamaterials with atomically sharp edges.^26^

Theoretical predictions of large values of dielectric functions of TMDs and related vdW materials include density functional theory (DFT) studies, which confirm exceptionally high values and the anisotropic nature of permittivity tensors in these materials.^27−30^ Theoretically, MoTe_2_, ReS_2_, and ReSe_2_ compounds were predicted to have the highest values of the in-plane dielectric function. Together, the experimental observations and theoretical predictions strongly motivate further development of all-TMD nanophotonics.^31^ The knowledge of optical constants of these materials is essential in this regard. However, these parameters are often not precisely known, the out-of-plane values are often assumed, and the response is measured within a too narrow spectral range. In addition, the quality of the extracted optical constants for any given TMD material strongly depends on the sample preparation,^11,32^ number of layers,^33,34^ and their lateral dimensions. The emerging field of all-TMD nanophotonics requires accurate knowledge of both in-plane and out-of-plane optical constants of various TMDs over a broad spectral range. Such knowledge would allow the exploration of their potential not only in the visible but also in the near-infrared nanophotonic and optoelectronic applications. Here, we present a library of optical constants of several TMD materials, obtained using a sensitive and accurate spectroscopic ellipsometry approach. The method accounts for sample and measurement imperfections, such as surface roughness, thickness nonuniformity, and spectrometer bandwidth and beam angular spread, ensuring a good level of consistency between all our results.

In this work, we have measured both in-plane and out-of-plane optical constants of high-quality mechanically exfoliated TMDs over a broad spectral range of 300–1700 nm using spectroscopic ellipsometry. Specifically, we investigated several important multilayer TMDs with large lateral dimensions, including semiconducting WS_2_, WSe_2_, MoS_2_, MoSe_2_, and MoTe_2_, as well as in-plane anisotropic ReS_2_ and WTe_2_ and metallic TaS_2_, TaSe_2_, and NbSe_2_ materials.^29,30^ Our experimentally obtained data reveal several interesting regimes, including high-index, anisotropy, and hyperbolicity, that may prove useful for future nanophotonic and optoelectronic applications.

## Results and Discussion

To extract the optical constants, we prepared all multilayer TMD flakes by mechanical exfoliation directly from high-quality bulk crystals (HQ-graphene) onto substrates. We note that in order to perform spectroscopic ellipsometry measurements with low uncertainty, the lateral dimensions of the TMD flakes are critical and must be larger than the beam spot during the measurements.

**Figure 1:**
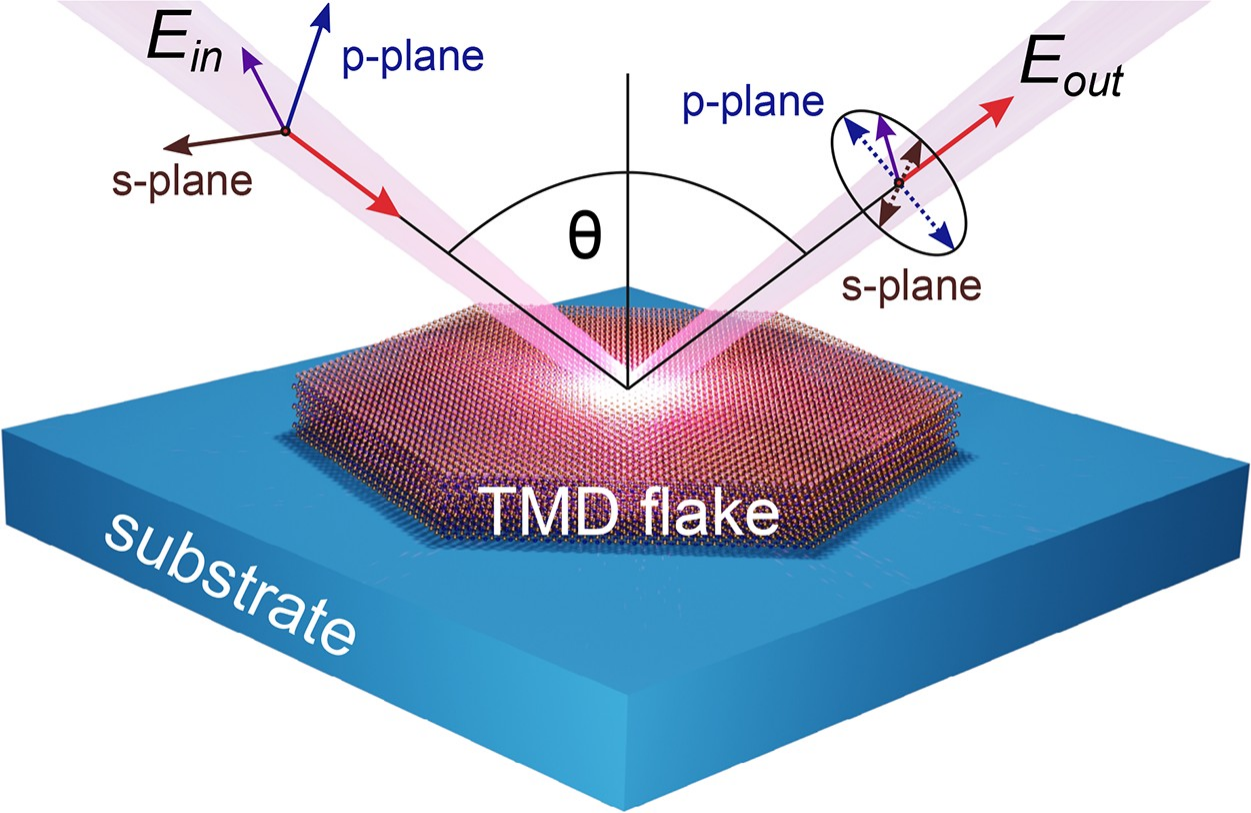
Schematic of the spectroscopic ellipsometry measurements on mechanically exfoliated multilayer TMDs on a substrate. The sample is illuminated by a collimated and polarized beam containing p- and s-components. Optical properties of the sample are retrieved from the change of the polarization state induced by the reflection from the TMD surface. The change is measured as a function of incidence angle θ and wavelength in a broad angular and spectral range.

**Figure 2:**
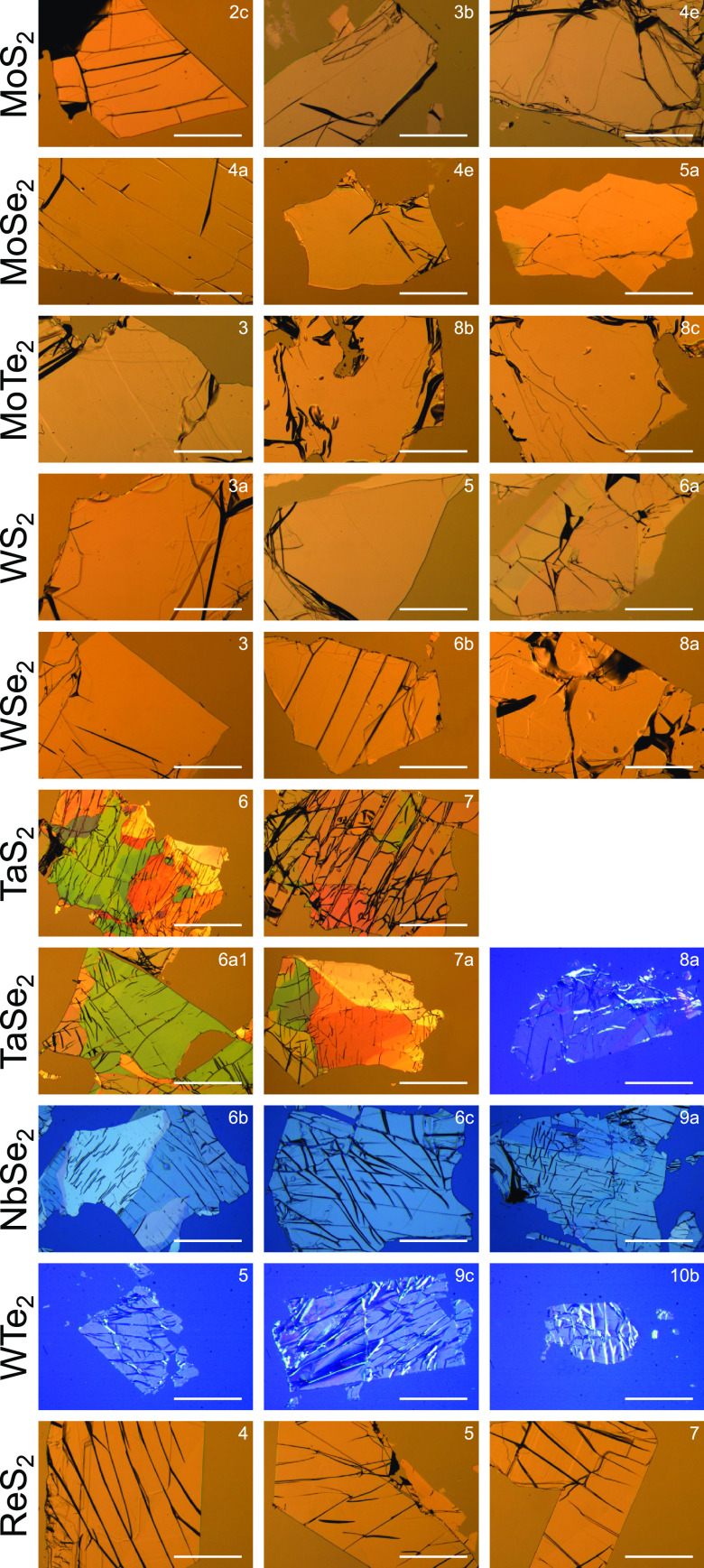
Exemplary images of exfoliated TMD flakes used for ellipsometric measurements. The first five rows show uniaxial semiconducting TMD flakes: MoS_2_, MoSe_2_, (2H)MoTe_2_, WS_2_, and WSe_2_, respectively. The next three rows contain uniaxial metallic TMD flakes: TaS_2_, TaSe_2_, and NbSe_2_. The last two rows contain the two characterized biaxial TMD flakes: metallic (though in the optical range the permittivity is positive) WTe_2_ and semiconducting ReS_2_, respectively. All semiconducting TMD flakes were placed directly on a Si substrate with a rough (diffusive) backside. In the case of metallic TMDs a thermally oxidized Si substrate was used (with a SiO_2_ thickness of 3 or 8.8 μm). The scale bar is 300 μm long and the same in every panel.

In this work, the spectroscopic ellipsometry measurements (see Figure 1 for scheme) were performed using a variable-angle spectroscopic ellipsometer with a dual rotating compensator design (VASE Woollam RC2) equipped with focusing probes to reduce the beam diameter to ∼300 μm. To obtain multilayer TMDs of sufficient lateral dimensions, all multilayer TMD flakes were mechanically exfoliated from bulk crystals, first, onto polydimethylsiloxane (PDMS) stamps using the Scotch-tape method. Subsequently, the partially transparent semiconducting flakes were transferred onto one-side-polished silicon substrates with a self-limiting natural oxide layer (∼1–3 nm) using the all-dry-transfer method^35^ with a few important concerns.^36^ The lossy and/or metallic TMD flakes were transferred onto silicon substrates with thermally grown SiO_2_ with nominal thicknesses of 3 or 8.8 μm. First, we chose the original bulk crystals carefully and exfoliated multilayers onto PDMS stamps only from large (at least a centimeter) homogeneous crystals using blue Scotch tape. Second, due to the thermoplastic properties of the PDMS film, the adhesion between the TMD flakes and PDMS slightly decreases at elevated temperature (here 60 °C). By exploiting this property, large multilayer TMDs with a relatively homogeneous thickness can be readily transferred onto a substrate for ellipsometric measurements. Thicknesses of the transferred TMD flakes were measured using a VEECO profilometer. For our study, we chose multilayer TMDs with thicknesses ranging from a few tens of nanometers to several microns. Exemplary TMD flakes are shown in Figure 2. These include semiconducting WS_2_, WSe_2_, MoS_2_, MoSe_2_, MoTe_2_, as well as biaxial ReS_2_ and WTe_2_ and metallic TaS_2_, TaSe_2_, and NbSe_2_, which are among the most promising TMDs for future nanophotonic and optoelectronic applications. After sample preparation, we performed spectroscopic ellipsometry measurements and analysis for all freshly prepared multilayers in a broad spectral range of 300–1700 nm in steps of 1 nm. The measurements were performed at multiple angles of incidence ranging from 20° to 75° in steps of 5°. For some flakes the maximum angle was reduced to ensure that the illumination spot was smaller than a TMD flake. The obtained dielectric tensor component data as a function of wavelength for all studied materials are provided in the Supporting Information (SI).

### Uniaxial Semiconductors

We begin our study by investigating multilayers of uniaxial TMD semiconductors, such as MoS_2_, MoSe_2_, MoTe_2_, WS_2_, and WSe_2_. These are one of the most well-studied TMD materials, especially in relation to their monolayers^2^ and vdW heterostructures.^37^ Recently, they have been also suggested as a promising high-index dielectric material platform for future nanophotonics.^10,14,28,31,38^ Here, we report both their in-plane and out-of-plane dielectric constants and compare our results to previously reported values.^9,10,27,28,39,40^

In general, ellipsometry measures changes of the polarization state of light upon reflection of an incident beam from a sample (Figure 1). The change is represented by two measured parameters, Ψ (psi) and Δ (delta), which correspond to the ratio of the reflection coefficients and the phase difference between the p- and s-polarization components of the incident beam. The approach described above assumes that no exchange between the polarization components occurs upon reflection from the sample. In the case of anisotropic samples, cross-polarization might occur; thus more complex analyses including general ellipsometry or Mueller matrix ellipsometry are required to address this issue. However, in the case of uniaxial anisotropic materials proper sample alignment results in canceling of all the off-diagonal components of the Mueller matrix, which simplifies the procedure to the standard ellipsometry. While this alignment is straightforward for TMD flakes due to their vdW nature, which dictates the alignment of the crystalline axes, the full Mueller matrix was measured to ensure this proper alignment (Figure S1–Figure S10).

To extract physical parameters such as the thickness or complex refractive index of a given material, an appropriate model describing the investigated structure has to be built. Parameters of interest are extracted by a simultaneous fit of the model parameters to the Ψ and Δ curves. Although the technique allows for optical characterization of a sample with thickness down to a monolayer, measurements of the anisotropic samples are challenging^41^ and require thick samples to ensure sufficient light interaction with in-plane and, especially, out-of-plane polarization components to sense the anisotropy. That is particularly difficult for samples with a high refractive index, because the angle of refraction at the sample–air interface is greatly reduced, which in turn hampers accurate determination of the out-of-plane components. This problem can be partially overcome by using an appropriate scheme of measurements and analysis that allows improving the sensitivity of the model (see Methods).

**Figure 3:**
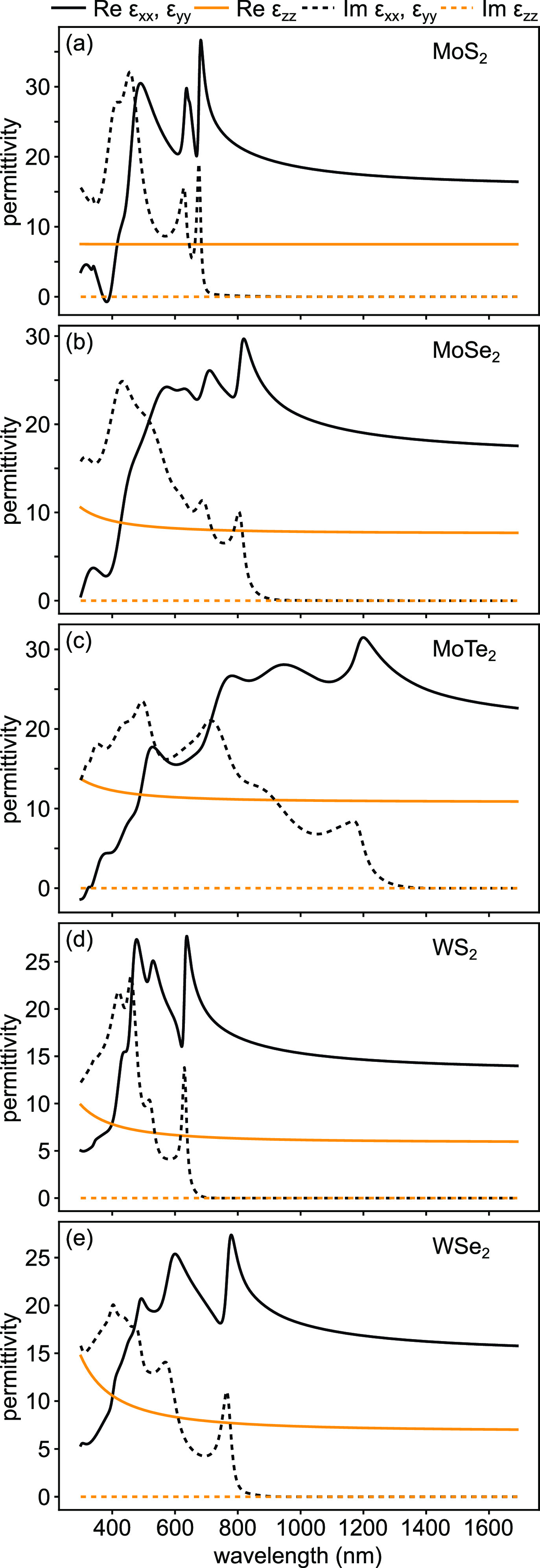
Permittivity of uniaxial semiconducting TMD flakes: (a) MoS_2_, (b) MoSe_2_, (c) MoTe_2_, (d) WS_2_, and (e) WSe_2_. Corresponding exemplary Mueller matrix measurements proving their uniaxial nature are plotted in Figures S1–S5.

In our experiments, the samples exhibiting transparency in the visible–near-infrared (vis–NIR) range are present in the form of TMD layers (with thickness varying from a few tens of nm to a few microns) mechanically exfoliated onto Si substrates with a native SiO_2_ layer. In the extraction procedure, we use a multisample analysis approach with the model containing a semi-infinite Si substrate with a native oxide and a layer of TMD with variable thickness. The optical constants of Si and SiO_2_ are taken from the CompleteEASE database, and their validity was confirmed by reference measurements of substrates next to the TMD flakes. The in-plane component of the complex refractive index is described by multiple Tauc–Lorentz dispersion model terms, while the out-of-plane component is described by a single resonance described by ε_*∞*_ and its ultraviolet (UV) position and amplitude (see Methods). In the analysis, both surface roughness and layer nonuniformity are taken into account, and the goodness of fit parameter, defined as the mean squared error (MSE), is minimized during the fitting procedure. The sensitivity of the technique to the anisotropic properties of the samples can be deduced from the asymmetry of the interference maxima in the Ψ curves occurring in the transparent regions of the samples. Moreover, a significant drop of MSE when the permittivity model is changed from isotropic to anisotropic indicates that this approach allows for extracting the out-of-plane components. Figure 3 shows the complex permittivities of the analyzed uniaxial semitransparent TMDs in the 300–1700 nm range (see Figures S11–S15 for quality of the fits to Ψ and Δ). The surface of some samples is nonuniform, cf. Figure 2, with wrinkles and folds that cannot be easily incorporated into roughness within the model. Since the surface features influence the UV region the most, we limit the data to 300 nm in order to minimize the uncertainty of the extracted dispersion curves.

**Figure 4:**
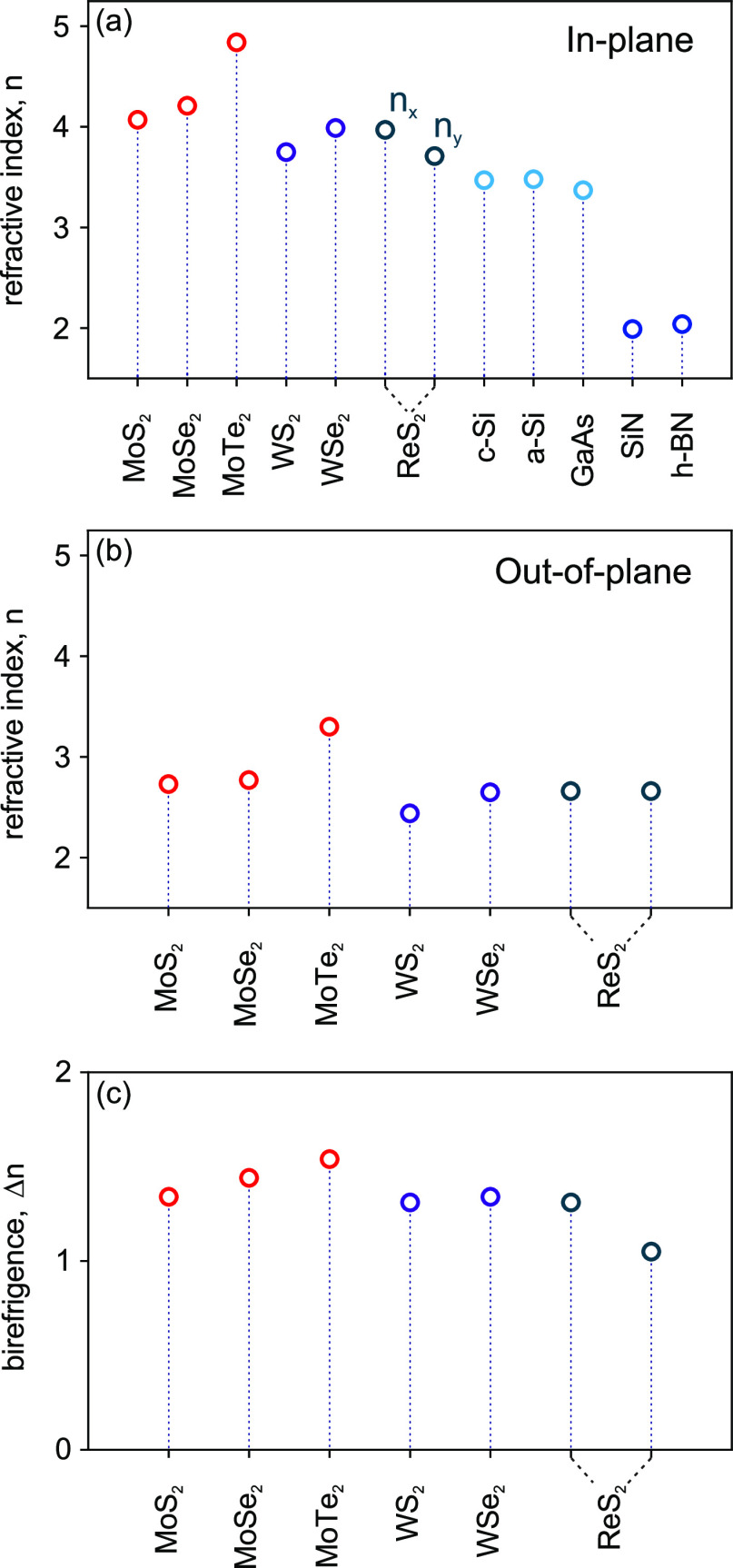
Comparison of extracted refractive indices of common TMDs at the telecom C band (1550 nm) with conventional semiconductors. (a) In-plane refractive index, (b) out-of-plane refractive index, (c) birefringence Δ*n* = *n*_∥_ – *n*_⊥_.

Extracted in-plane (*n*_∥_, here ) and out-of-plane  refractive indices at 1550 nm for semiconducting TMDs and their optical anisotropy (Δ*n* = *n*_∥_ – *n*_⊥_) are displayed in Figure 4. Overall, multilayer TMDs exhibit higher refractive indices at 1550 nm, in comparison to conventional high-index dielectrics, such as c-Si (∼3.47),^42^ a-Si (∼3.48),^43^ and GaAs (∼3.37).^44^ Our data in Figure 4a reveal a few interesting trends. First, we observe higher index values among Mo-based TMDs, ∼4.07 (MoS_2_), ∼4.21 (MoSe_2_), and ∼4.84 (MoTe_2_), when compared to W-based WS_2_ (∼3.75) and WSe_2_ (∼3.99) materials. Second, these data also reveal that the refractive index increases in the following order: . This observation agrees well with theoretically predicted results from an earlier DFT study.^27^ In addition to high index, it is worth mentioning that optical losses for all studied materials shown in Figure 3 are negligible in the near-infrared range. This opens a possibility of using multilayer TMDs for low-loss nanophotonics, including waveguides and high quality factor resonators.^31,45^

In the visible spectral range, the imaginary parts of permittivities exhibit pronounced resonances corresponding to A-, B-, and C-excitons (Figure 3). For instance, in Figure 3, the in-plane imaginary parts of permittivities show clear A-exciton resonances at ∼676 nm (1.835 eV in MoS_2_), ∼803 nm (1.544 eV in MoSe_2_), ∼1167 nm (1.062 eV in MoTe_2_), ∼629 nm (1.972 eV in WS_2_), and ∼764 nm (1.624 eV in WSe_2_), respectively. The extracted values of A-exciton resonances are in reasonable agreement with previous reports.^11,39,40,46,47^

An important feature of all studied multilayer TMDs is their large birefringence Δ*n*. Figure 4c shows that TMDs exhibit Δ*n* ≥ 1.3 due to smaller out-of-plane refractive indices ranging from *n*_⊥_ = 2.44 (WS_2_) to 3.3 (MoTe_2_) (see Figure 4b), in comparison to their in-plane indices (see Figure 4a). MoTe_2_ shows the largest birefringence of Δ*n* ≈ 1.54 among uniaxial semiconducting TMDs, whereas other materials MoS_2_, MoSe_2_, WS_2_, and WSe_2_ exhibit birefringence values of Δ*n* = ∼1.34, ∼1.44, ∼1.31, and ∼1.34, respectively. The birefringence qualitatively follows the same  and  order as in-plane refractive indices. It should also be noted that the obtained birefrigence values in TMDs are 7–8 times larger than common anisotropic materials, such as yttrium orthovanadate and rutile TiO_2_, which exhibit birefringences of Δ*n* ≈ 0.2–0.3.^48^ Our data are in a good agreement with previous experimental and theoretical reports on birefringence.^7,9,27^ For instance, our result for MoS_2_, Δ*n* ≈ 1.34, at 1550 nm is in agreement with previously obtained experimental values of Δ*n* ≈ 1.4 (at 1530 nm, extracted by scattering-type scanning near-field optical microscopy (s-SNOM)^7^) and Δ*n* ≈ 1.5 (in the infrared, extracted by spectroscopic ellipsometry^9^). However, due to the lack of reported birefringence data for a broader range of TMD materials, it is difficult to perform a comprehensive comparison. Our study partially fills this gap and, thus, contributes to the database of available optical constants of TMD materials, which may prove useful for the development of future all-TMD nanophotonic applications. Additionally, a combination of high index (*n* ≳ 4), low loss, and large birefringence (Δ*n* ≳ 1.4) in the near-infrared range makes multilayer TMDs a promising material platform for exploring photonic surface waves, e.g., Dyakonov^49,50^ and Zenneck^8^ surface waves.

### Biaxial Semiconductor

**Figure 5:**
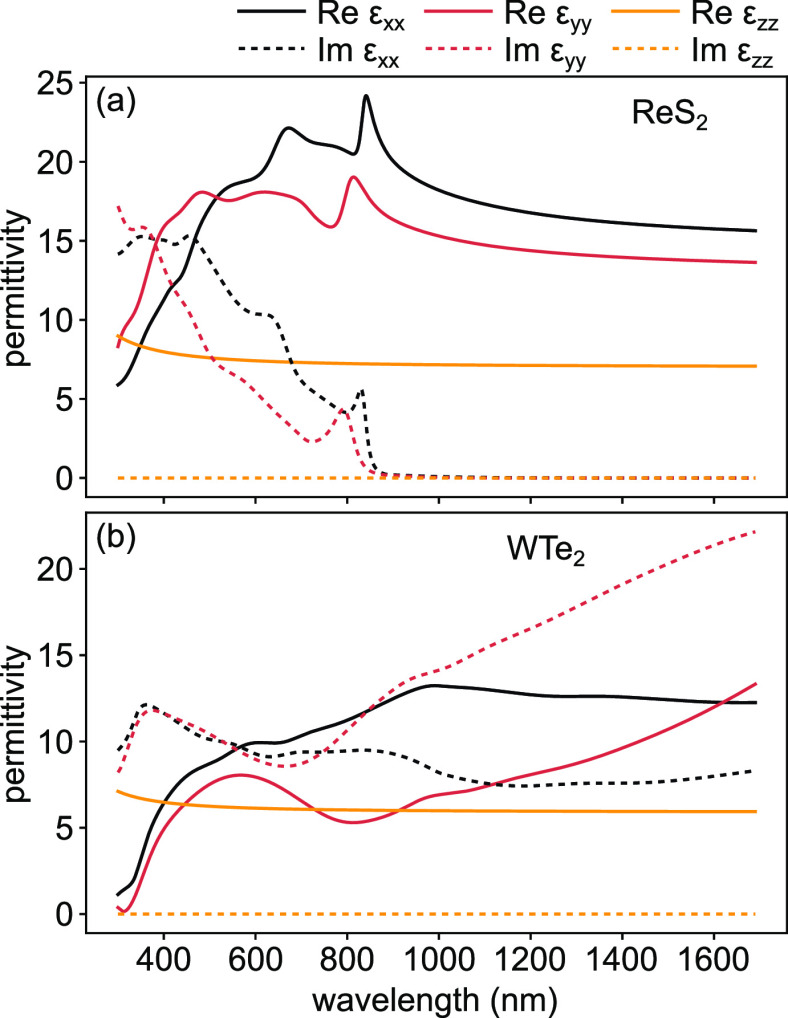
Permittivity of biaxial materials: (a) semiconducting ReS_2_ and (b) lossy (metallic at low frequencies) WTe_2_ TMD flakes. Corresponding exemplary Mueller matrix measurements in Figure S7 and Figure S6 show their biaxial nature.

We now turn to the biaxial semiconductor ReS_2_. The in-plane anisotropy of this material stems from the formation of covalent Re–Re bonds and correspondingly the 1T″-phase it adopts.^51^ This material, as well as its close relative ReSe_2_, has recently been predicted by DFT to have one of the highest permittivities in the visible–near-infrared spectral range (*n* > 5).^28^ Here, we report experimentally measured optical constants of ReS_2_. Performing ellipsometry on such a material is more challenging than for uniaxial TMDs from the measurement as well as analysis perspective. In-plane anisotropy requires rotation of the sample during the measurement to extract Euler angles of the material’s crystallographic structure. This, in turn, requires that the lateral size of a ReS_2_ flake should be larger than the beam spot for all in-plane directions. However, due to in-plane anisotropy, ReS_2_ tends to shear-off along the *b*-axis,^52^ which makes exfoliation of large symmetric flakes extremely difficult. We were, however, able to prepare reasonably large ReS_2_ samples with some folds, which do not interfere with our measurements (Figure 2). An additional difficulty is that interaction of polarized light with a ReS_2_ sample leads to a cross-polarization effect; thus Ψ and Δ lose their meaning and the generalized ellipsometry, or more general Mueller matrix ellipsometry, is required. To ensure sensitivity of the methods to both in-plane and out-of-plane components, we performed a multisample analysis with ReS_2_ thicknesses ranging from 200 nm to approximately 600 nm and at least two rotation angles with other measurement parameters being the same as for uniaxial semiconducting TMDs above. It is worth mentioning that due to problems with exfoliation, some ReS_2_ flakes are characterized by a terrace-like surface morphology and are not uniform in thickness. Thus, in the analysis, the data acquired for each rotation angle are treated as for a separate sample and its thickness (for every in-plane rotation angle) is a free fitting parameter. However, despite the presence of terraces, the crystalline axes of each flake remain invariant, and thus the fitted orientation (in-plane rotation) angles are consistent with the rotation angles during measurements. The in-plane permittivities ε_*xx*_ and ε_*yy*_ are described by multiple Tauc–Lorentz dispersion models, while the out-of-plane component is described by a single resonance. The experimentally obtained permittivity for ReS_2_ is shown in Figure 5a, while the fidelity of the fits is in Figure S17.

Here, we extracted two *n*_∥_ (*n*_*xx*_ and *n*_*yy*_) and one *n*_⊥_ (*n*_*zz*_) refractive index of ReS_2_ at 1550 nm from the measured permittivity, which yielded *n*_*xx*_ = 3.97, *n*_*yy*_ = 3.71, and *n*_*zz*_ = 2.66, respectively (Figure 4a,b). ReS_2_ exhibits birefringence values of Δ(*n*_*xx*_ – *n*_*zz*_) ≈ 1.31 and Δ(*n*_*yy*_ – *n*_*zz*_) ≈ 1.05 (Figure 4c). The obtained Δ(*n*_*yy*_ – *n*_*zz*_) ≈ 1.05 is smaller than the birefringence of other TMDs. However, our data show that ReS_2_ possesses an in-plane birefringence of Δ(*n*_*xx*_ – *n*_*yy*_) ≈ 0.26 at 1550 nm, in addition to its out-of-plane anisotropy. This suggests that ReS_2_ could be an interesting material for next-generation photonics due to its both in-plane and out-of-plane anisotropic properties (Re(ε_*xx*_) ≠ Re(ε_*yy*_) ≠ Re(ε_*zz*_) ≠ Re(ε_*xx*_)), together with high index (≥3.7) and low loss in the telecom range. Moreover, a recent study reports a light-induced phase transition in mono- and bilayers of ReS_2_, which could be an additional benefit for ReS_2_-based nanophotonic applications, if such a process could be extended to multilayer ReS_2_.^53^ Finally, it is noteworthy that in the visible spectral range the in-plane anisotropic permittivity of ReS_2_ reveals two-orthogonal A-excitons at ∼792 nm (1.566 eV in Im(ε_*yy*_)) and ∼830 nm (1.495 eV in Im(ε_*xx*_)), respectively (Figure 5a).

### Metallic and Hyperbolic (Uniaxial and Biaxial)

The third group of samples investigated in the experiment are TMDs that exhibit absorption in the whole measured wavelength range that comes from the metallic response and/or additional interband transitions.^29^ Due to a lack of sharp features in Ψ and Δ spectra when placed directly on a reflective substrate, they require a special scheme of measurements to ensure sufficient interaction of light with the samples and uniqueness of the ellipsometric models. This additional requirement is obtained by using a few-micron-thick thermally grown SiO_2_ layer on top of a Si substrate. When a thin semitransparent absorbing TMD flake is deposited on such a support, interference in the SiO_2_ layer yields a distinct modulation of the ellipsometric signal, whose contrast depends on the thickness and extinction coefficient of the TMD layer. This so-called interference approach was introduced by Hilfiker et al.,^54^ showing great improvement in the sensitivity of ellipsometric models applied to absorbing materials.

In our experiments semitransparent flakes of thicknesses ranging from ∼50 to ∼400 nm were exfoliated onto a 3 or 8.8 μm thick thermally grown SiO_2_ layer on a Si substrate. The measurements with the use of standard ellipsometry were carried out for the same wavelength and incidence angle range as for the previous samples (cf. Figures S8–S10 for Mueller matrix measurements confirming appropriate alignment of the uniaxial samples). In the case of biaxial WTe_2_ the significant difference with respect to the details above was the need to perform in-plane rotation of the sample identically to ReS_2_. The anisotropic materials were modeled with Tauc–Lorentz and as needed Drude functions for both in-plane and out-of-plane components. The fitting procedure for these two types of materials is described in the Methods section.

**Figure 6:**
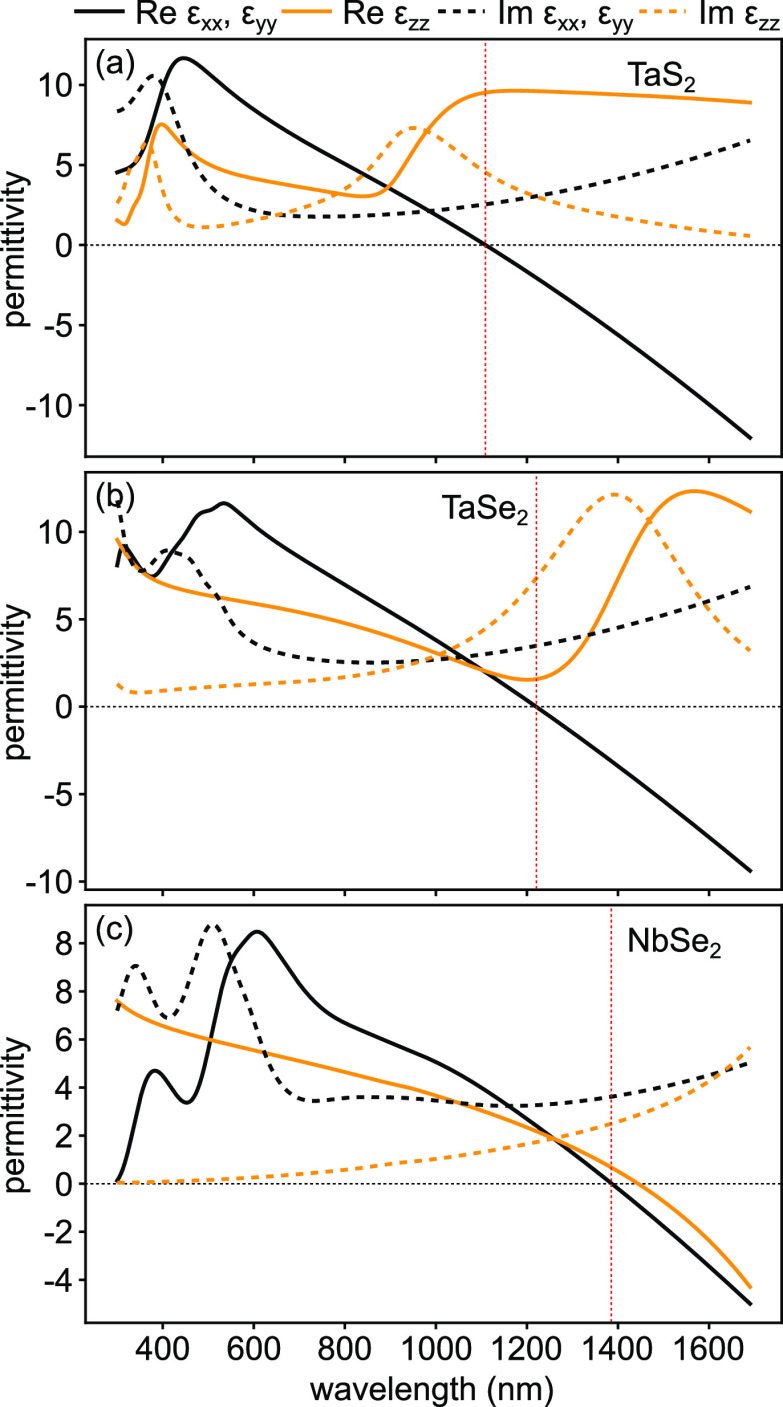
Permittivity of uniaxial metallic TMD flakes: (a) TaS_2_, (b) TaSe_2_, and (c) NbSe_2_. See Figures S8–S10 for Mueller matrix measurements confirming their uniaxial properties and proper alignment. In all panels, horizontal black lines separate positive and negative permittivities, while vertical red lines show the wavelengths at which the in-plane permittivities cross zeros. In the measured spectral range, wavelengths to the right of the red lines correspond to regions of hyperbolicity for TaS_2_ and TaSe_2_, while for NbSe_2_ hyperbolicity is not observed due to the appearance of a Reststrahlen band for wavelengths above ∼1400 nm in Re(ε_*zz*_) (see text).

We now study uniaxial metallic TMDs, e.g., TaS_2_, TaSe_2_, and NbSe_2_, whose permittivities obtained in our measurements are shown in Figure 6. Interestingly, the materials exhibit both dielectric and metallic responses in the studied spectral range, as indicated by a change of the sign of the values of the in-plane Re(ε) from positive to negative around 1100 to 1400 nm, depending on the material. Specifically, the plasma frequencies of the in-plane Re(ε) of Ta-based TMDs TaS_2_ and TaSe_2_ are respectively at ∼1110 nm (∼1.11 eV) and ∼1217 nm (∼1.01 eV), as illustrated in Figure 6a,b. However, their out-of-plane Re(ε_*zz*_) remain positive, showing only a dielectric response over the entire studied spectral range. This suggests that TaS_2_ and TaSe_2_ exhibit a natural hyperbolic response at frequencies below their corresponding plasma frequencies, which could be useful for TMD plasmonic applications.^29,30^

On the other hand, Figure 6c shows that both in-plane and out-of-plane components of Re(ε) of NbSe_2_ are positive for wavelengths shorter than ∼1390 nm, suggesting that NbSe_2_ may behave like an out-of-plane anisotropic dielectric in the visible spectrum. Conversely, the real parts of the diagonal dielectric tensor become negative at longer wavelengths and retain the sign until the red edge of our measurement range. Furthermore, above ∼1200 nm NbSe_2_ is only weakly anisotropic. The negative sign of the in-plane dielectric function is associated with a free-electron response just like for Ta-based metallic TMDs and indicates an in-plane plasma frequency of ∼0.89 eV. However, the negative dielectric function in the out-of-plane direction is not associated with a free-electron Drude-like response. Rather it is due to a high oscillator strength of a Lorentz-like response, which leads to the appearance of a Reststrahlen-like band for wavelengths above ∼1400 nm. The out-of-plane dielectric function should therefore become positive again at longer wavelengths; however, this occurs outside of our measurement range (>1700 nm).

In contrast to the above three materials, WTe_2_ is an in-plane anisotropic TMD like ReS_2_, but is metallic and hyperbolic (the plasma frequency of this material is, however, much smaller than that of other metallic TMDs studied in this work, so in the 300–1700 nm spectral range it appears to have a dielectric-like response). Recently, hyperbolic dispersion in WTe_2_ was demonstrated experimentally in the infrared region (∼500 cm^–1^) using far-field absorption measurements.^55^ Later, a new hyperbolic regime in the near-infrared (∼1 eV) was theoretically predicted in monolayer WTe_2_ due to band-nested anisotropic interband transitions.^56^ However, such hyperbolicity of WTe_2_ in the near-infrared becomes weaker and disappears as the layer number increases from monolayer to bulk. Indeed, Figure 5b shows the experimentally here-obtained permittivity from multilayer WTe_2_. As expected, the real part of the permittivity Re(ε) shows no sign of hyperbolic behavior in the studied spectral range. At the same time, we observed an interesting anisotropic behavior in the narrow spectral region around 800 nm, where Re(ε_*xx*_) > Re(ε_*zz*_) > Re(ε_*yy*_). Such behavior is uncommon and is not observed in other TMDs studied here. Moreover, WTe_2_ possesses relatively large in-plane birefringence ranging from a Δ(*n*_*xx*_ – *n*_*yy*_) of ∼0.2 (at 510 nm) to ∼1.12 (at 880 nm). The maximum obtained Δ(*n*_*xx*_ – *n*_*yy*_) of ∼1.12 (WTe_2_) is substantially larger than the in-plane birefringence of ReS_2_ (∼0.26). We note, however, that the in-plane losses are relatively large in the entire studied spectral range.

## Conclusion

In conclusion, we have experimentally measured both in-plane and out-of-plane optical constants of mechanically exfoliated TMD multilayers using spectroscopic ellipsometry over a broad spectral range of 300–1700 nm. Our measurements include several semiconducting, WS_2_, WSe_2_, MoS_2_, MoSe_2_, and MoTe_2_ as well as in-plane anisotropic ReS_2_ and WTe_2_ and metallic TaS_2_, TaSe_2_, and NbSe_2_ materials. The extracted parameters demonstrate a combination of several remarkable optical properties, such as a high index (*n* up to ∼4.84 for MoTe_2_), significant anisotropy (*n*_∥_ – *n*_⊥_ ≈ 1.54 for MoTe_2_), and low absorption in the near-infrared region. Moreover, metallic TMDs show potential for a combined plasmonic–dielectric behavior and hyperbolicity, as their plasma frequencies occur in the ∼1000–1300 nm range depending on the material. The knowledge of dispersive and anisotropic optical constants of these vdW materials opens new possibilities for the future development of all-TMD nanophotonics.

## Methods

### Sample Preparation

All TMD flakes, including semiconducting and uniaxial WS_2_, WSe_2_, MoS_2_, MoSe_2_, and (2H)MoTe_2_, hyperbolic and metallic (2H)TaS_2_, TaSe_2_, and NbSe_2_, and biaxial ReS_2_ and WTe_2_, were mechanically exfoliated from bulk crystals (HQ-graphene) onto PDMS stamps using the Scotch-tape method and then transferred onto substrates using the all-dry-transfer method.^35,36^ For spectroscopic ellipsometry measurements, the lateral dimensions of TMD flakes should be larger than the beam size. To obtain sufficient dimensions of TMD flakes, we modified the previously developed method with a few important concerns.

First, we chose the starting bulk crystals carefully and exfoliated multilayers onto PDMS stamps only from large crystals (a centimeter at the least) with a homogeneous surface using blue Scotch tape. Second, due to the thermoplastic properties of the PDMS film, adhesion between TMD flakes and PDMS slightly decreases at the elevated temperature of 60 °C. By exploiting this advantage, large multilayer TMDs with relatively homogeneous thicknesses can be readily transferred onto a substrate for ellipsometric measurements. The thicknesses of the transferred TMD flakes were measured using a VEECO profilometer, and we chose multilayer TMDs with thicknesses ranging from a few tens of nanometers to several microns. The minimum lateral size of uniaxial TMD flakes investigated in this study is at least 300 μm in one direction (beam width) and at least 700 μm in the other to facilitate measurements at angles of incidence of up to (and a minimum of) 65°. For biaxial TMDs, ReS_2_ and WTe_2_, both orthogonal in-plane directions need to be not smaller than 400 μm to enable measurements up to (at least) 45° and larger if possible. For ellipsometric measurements of semiconducting TMDs, one-side-polished silicon substrates with a self-limiting natural oxide layer (1–3 nm) were used. Metallic TMDs lack sharp excitonic features in Ψ and Δ spectra. Therefore, in order to perform high-quality measurements of metallic TMDs and to obtain sharp interference features in the ellipsometric spectra, semitransparent metallic TMD flakes of TaS_2_, TaSe_2_, NbSe_2_, and WTe_2_ were transferred onto silicon substrates with a 3 or 8.8 μm thick thermally grown SiO_2_ layer.

### Variable-Angle Spectroscopic Ellipsometry

Ellipsometric measurements were carried out using a Woollam RC2 dual rotating compensator (DRC) ellipsometer with a vertical auto angle stage. It allows for measurement of the full Mueller matrix, which is essential for analysis of biaxial samples and helpful for verifying that the uniaxial samples are correctly set up in the ellipsometer’s coordinates. Another feature that is accessible in the DRC architecture is the depolarization factor, which allows for measurement and modeling of a sample’s imperfections, such as thickness nonuniformity or the influence of a device’s parameters/limitations such as detector bandwidth or angular spread of the beam. Due to the small lateral dimensions of TMD flakes, they are measured using focusing probes, which reduce the light beam to a 300 μm spot at normal incidence. The configuration of the ellipsometer with mounted focusing probes does not allow for transmission measurements; thus samples were prepared and measured in reflection up to a wavelength of 1700 nm and the full accessible angle range from 20° to 75° depending of the size of the TMD flake. Modeling and fitting were done with the use of CompleteEASE v6.61.

### Fitting of Ellipsometric Data

The investigated samples exhibit three types of optical responses, which require different strategies in building appropriate models. The Ψ curves of uniaxial semitransparent TMD flakes exhibit interference maxima in the transparent regions. Proper fitting in these spectral ranges determines with very good accuracy both the thickness and the real part of the dielectric function, yielding a good starting point for further modeling of the data in the remaining spectral regions. For this type of samples we proceeded as follows:

- In the first step, an isotropic model is used and the transparent region is described by a Cauchy model, *A* + *B*/λ^2^, where the parameters such as layer thickness, *A*, and *B* are extracted by the Levenberg–Marquardt algorithm after fitting the model to the Ψ and Δ curves.
- In the next step, the Cauchy model is converted to the Kramers–Kronig consistent B-spline curves with their subsequent expansion to the whole wavelength range. The B-spline function approximates the fitting curves with basic functions with their argument (photon energy, eV) equally spaced. We used a 0.05 eV step in the whole energy range except where Ψ exhibits sharp or anomalous behavior corresponding to, for example, exciton bands in the dielectric function.
- In the third step, the isotropic model is converted to an anisotropic one and the out-of-plane component is described by a single UV resonance. After minimization of the MSE, the B-spline model is parametrized by a general oscillator model with the use of Tauc–Lorentz oscillators.
- The whole procedure is initially done for the thickest samples, and after parametrization, the fitting procedure is repeated in a multisample analysis, leading to a complex diagonal permittivity tensor, which is common for all samples.

The lack of a transparent region for the metallic TMD samples necessitates some changes to the above-described procedure. First of all, use of the interference approach requires a more complicated model taking into account the transparent interference layer as well as the additional interlayer present at the Si and SiO_2_ interface. The Si substrate with thermal SiO_2_ was characterized prior to the final measurements, and their extracted parameters were fixed in the initial stage of modeling.

- The first step consists of fitting an isotropic Kramers–Kronig consistent Bi-spline function to the data for the thinnest samples exhibiting the most pronounced interference maxima. The thickness of the TMD layer is fixed during the fitting procedure, and their values are taken from profilometric measurements.
- In the next step, the isotropic model is converted to an anisotropic one and the fitting procedure is repeated. For final improvement of the results, roughness and sample and machine imperfections are taken into account.
- In the last step the multisample analysis is carried out and the final Bi-spline model is parametrized with Tauc–Lorentz and Drude functions.

Evaluation of the models is based on minimization of the MSE, the correlation matrix, and the uniqueness test. In the case of biaxial samples the first and second procedures were used for respectively ReS_2_ and WTe_2_ with a modification that involved using a biaxial model instead of a uniaxial model.

### Ellipsometric Model Equations

The general model used to describe the optical properties of the studied TMDs is given by the following equation:
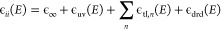
where ε_*ii*_(*E*) is one of the diagonal elements of the complex dielectric tensor as a function of photon energy, *E*, in eV with the subscript *ii* being *xx* or *yy* for the in-plane components or *zz* for the out-of-plane component. The four terms on the right-hand side are as follows:

- ε_*∞*_ is the real part of the permittivity at infinite frequency.
- ε_*uv*_(*E*) is equivalent to a Lorentz oscillator with zero broadening and position in the UV. Such resonances are positioned outside the measured spectral range and are applied to take into account absorption that occurs at higher energies than available in the experiment, thus influencing only the real part (specifically, its dispersion) of the permittivity in the measured spectral range. The UV resonance is defined as

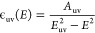
where the permittivity ε_uv_ is centered at energy *E*_uv_ and *A*_uv_ is the amplitude of the oscillator in eV^2^.

- The third term is a sum of Tauc–Lorentz complex functions with the imaginary part of the *n*th element being

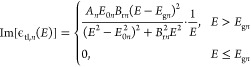
where *E*_0*n*_ is the center energy, *E*_g*n*_ the band gap energy of the oscillator, *B*_r*n*_ the broadening of the oscillator, and *A*_*n*_ the amplitude of the oscillator. The real part is calculated via the Kramers–Kronig transformation.

- The last term is the Drude function describing the electromagnetic response of a free-electron gas in conductive materials given by

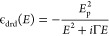
where *E*_p_ is the plasma energy and Γ is the damping.

## Supporting Information Available

Figures S1–S10, exemplary Mueller matrices for all materials; Figures S11–S20, agreement between measurements and fitted models; Tables S1 and S2, summary of other fitting parameters; Note S1, uncertainty of optical parameters accompanied by Figures S21–S23 (PDF)
Text files with the diagonal permittivity tensors plotted in Figure 3, Figure 5, and Figure 6 (ZIP)

## Notes

The raw ellipsometric data along with fitted models in both the proprietary format of the CompleteEASE software (Woolam) and as plain text files as well as plain text files with fitted permittivities are available at 10.5281/zenodo.6205431.
