# Supplementary material for: Optical Constants of Several Multilayer Transition Metal Dichalcogenides Measured by Spectroscopic Ellipsometry in the 300–1700 nm Range: High Index, Anisotropy, and Hyperbolicity
Source: ACS Photonics. 2022 Jun 7;9(7):2398–407. doi: 10.1021/acsphotonics.2c00433 (PMC9306003; doi:10.1021/acsphotonics.2c00433)
Supplement: Supplementary file 1 — ph2c00433_si_001.pdf [file ph2c00433_si_001.pdf]

Supporting Information

Optical constants of several multilayer transition metal dichalcogenides measured by spectroscopic ellipsometry in the 300-1700 nm range: high-index, anisotropy, and hyperbolicity

Battulga Munkhbat,[1,2,*] Piotr Wróbel,[3,*] Tomasz J. Antosiewicz,[3,1,†] and Timur O. Shegai[1,‡]

[1]*Department of Physics, Chalmers University of Technology, 412 96, Gothenburg, Sweden*
[2]*Department of Photonics Engineering, Technical University of Denmark, 2800 Kongens Lyngby, Denmark*
[3]*Faculty of Physics, University of Warsaw, Pasteura 5, 02-093 Warsaw, Poland*
**These authors contributed equally to this work.*
[†]*email: tomasz.antosiewicz@fuw.edu.pl*
[‡]*email: timurs@chalmers.se*

Contents

Figures – Examples of raw experimental data and ellipsometric fits 2

Tables of ellipsometric models' parameters 11
S1. Comparison of ellipsometric fitting parameters for uniaxial TMDs 11
S2. Comparison of ellipsometric fitting parameters for bianisotropic TMDs 12

Supporting Notes 12
S1. Uncertainty of optical constants 12

Figures – Examples of raw experimental data and ellipsometric fits

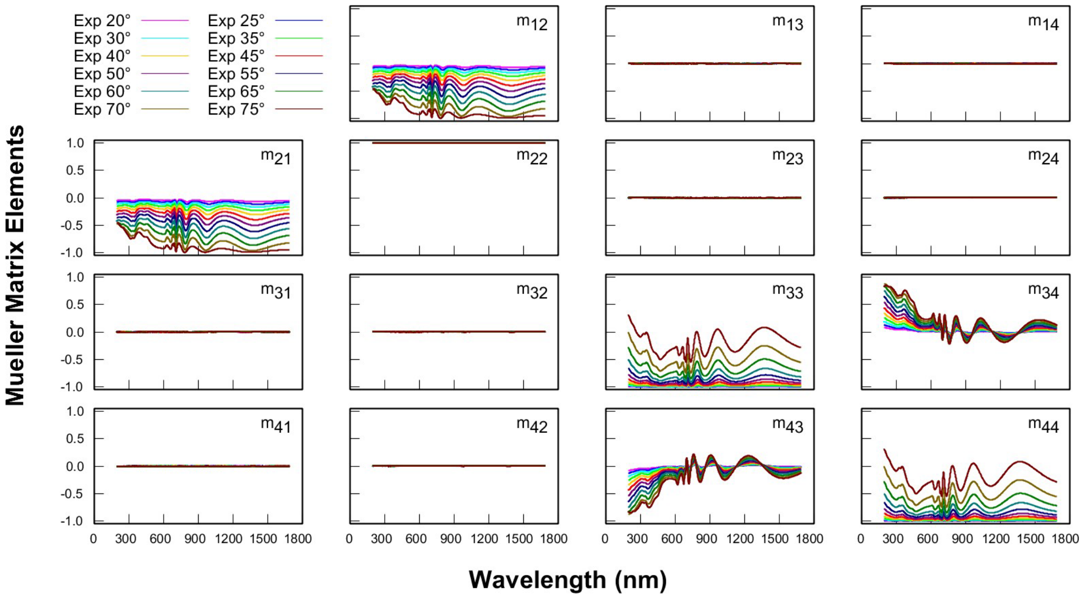

Figure S1: Mueller Matrix elements for a selected MoS_2 flake on a silicon substrate. Note that the zeros in the off-diagonal 2×2 sub-blocks prove the uniaxial nature of this material.

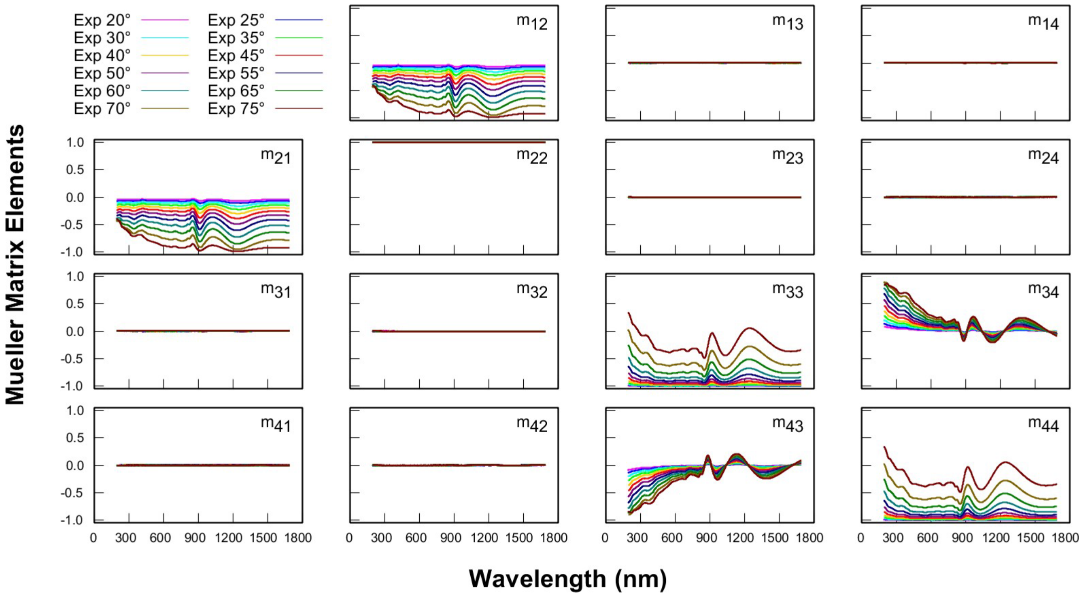

Figure S2: Mueller Matrix elements for a selected $MoSe_2$ flake on a silicon substrate. Note that the zeros in the off-diagonal 2×2 sub-blocks prove the uniaxial nature of this material.

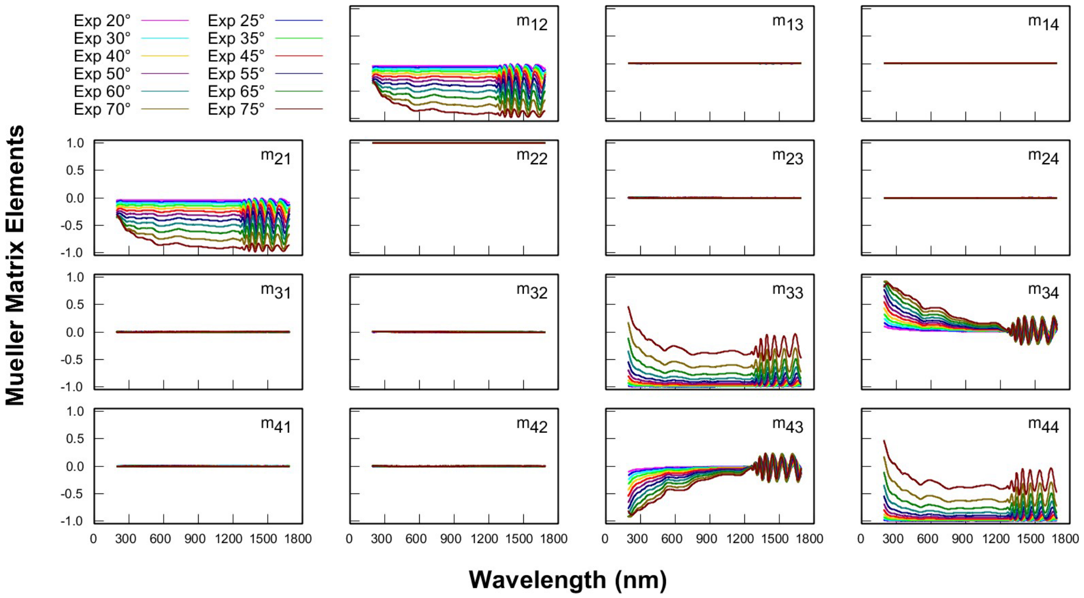

Figure S3: Mueller Matrix elements for a selected $MoTe_2$ flake on a silicon substrate. Note that the zeros in the off-diagonal 2×2 sub-blocks prove the uniaxial nature of this material.

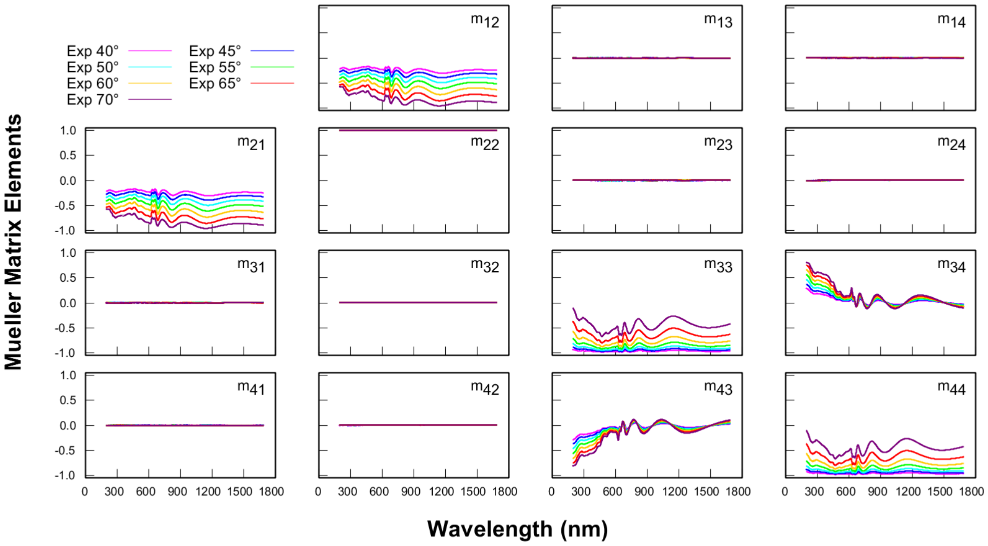

Figure S4: Mueller Matrix elements for a selected WS_2 flake on a silicon substrate. Note that the zeros in the off-diagonal 2×2 sub-blocks prove the uniaxial nature of this material.

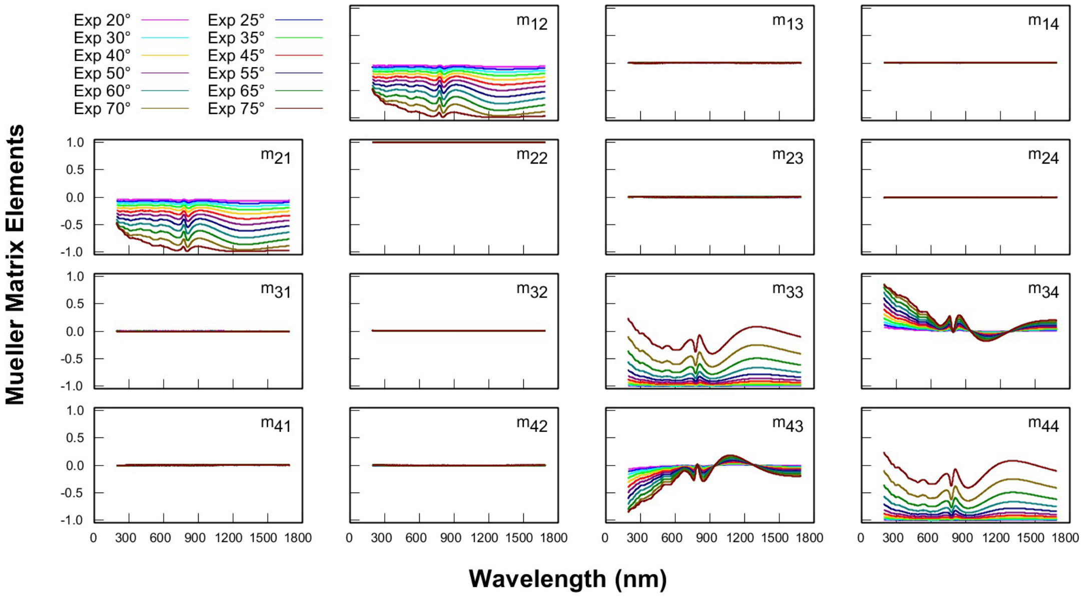

Figure S5: Mueller Matrix elements for a selected WSe_2 flake on a silicon substrate. Note that the zeros in the off-diagonal 2×2 sub-blocks prove the uniaxial nature of this material.

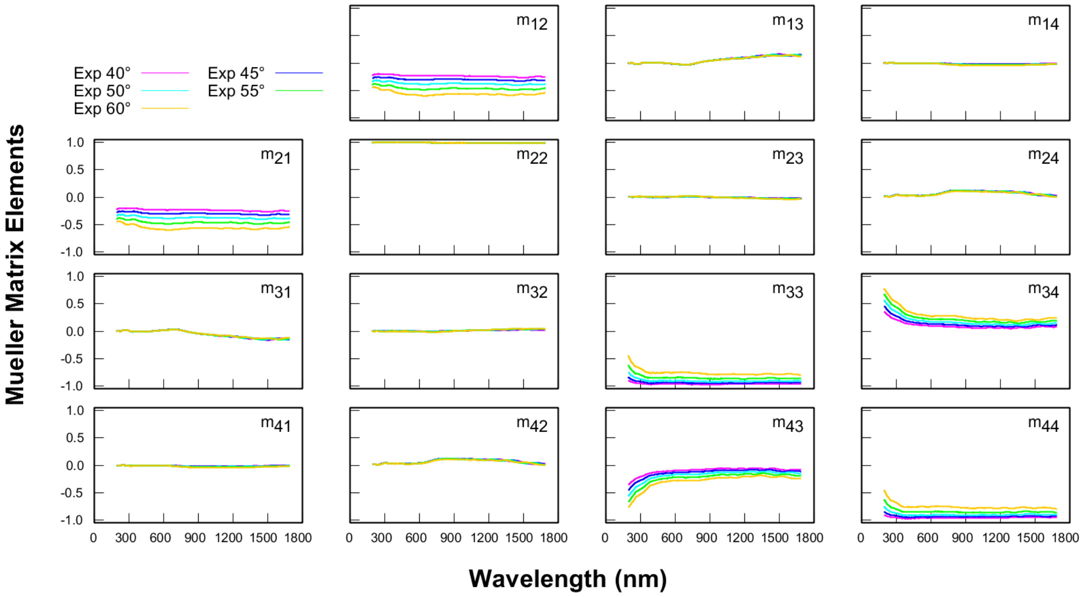

Figure S6: Mueller Matrix elements for a selected WTe_2 flake on a silicon substrate. Note that the off-diagonal 2×2 sub-blocks show the bianisotropic nature of this material.

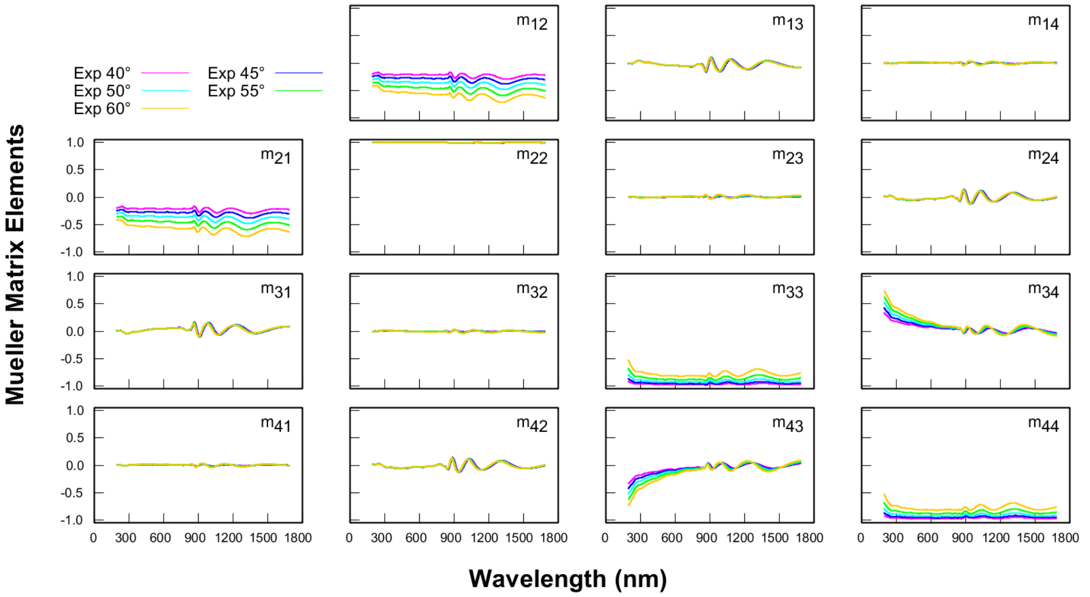

Figure S7: Mueller Matrix elements for a selected ReS_2 flake on a silicon substrate. Note that the off-diagonal 2×2 sub-blocks show the bianisotropic nature of this material.

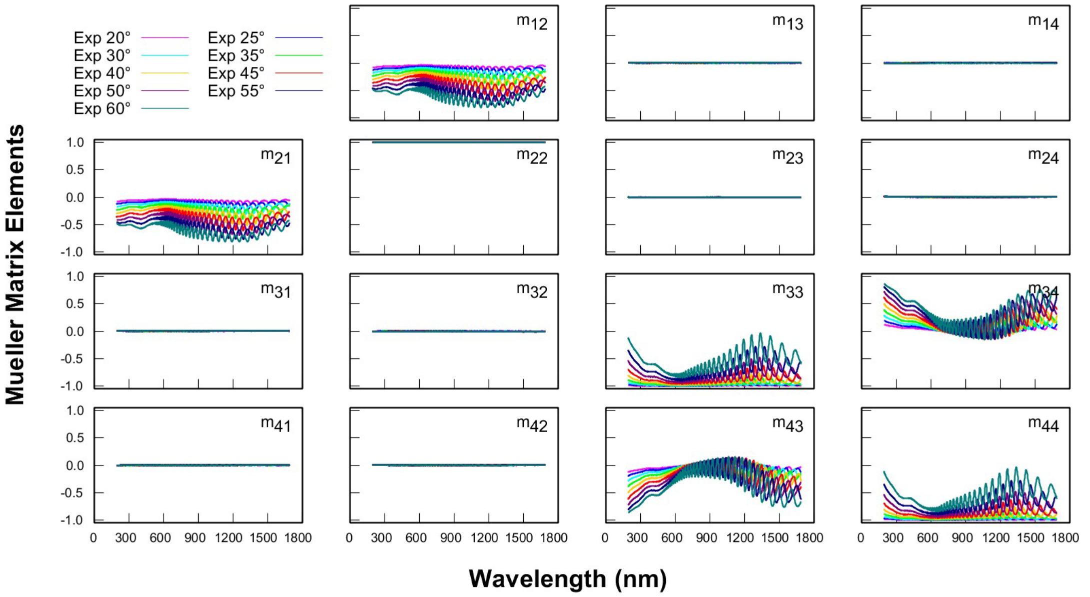

Figure S8: Mueller Matrix elements for a selected $NbSe_2$ flake on a silicon substrate. Note that the zeros in the off-diagonal 2×2 sub-blocks prove the uniaxial nature of this material.

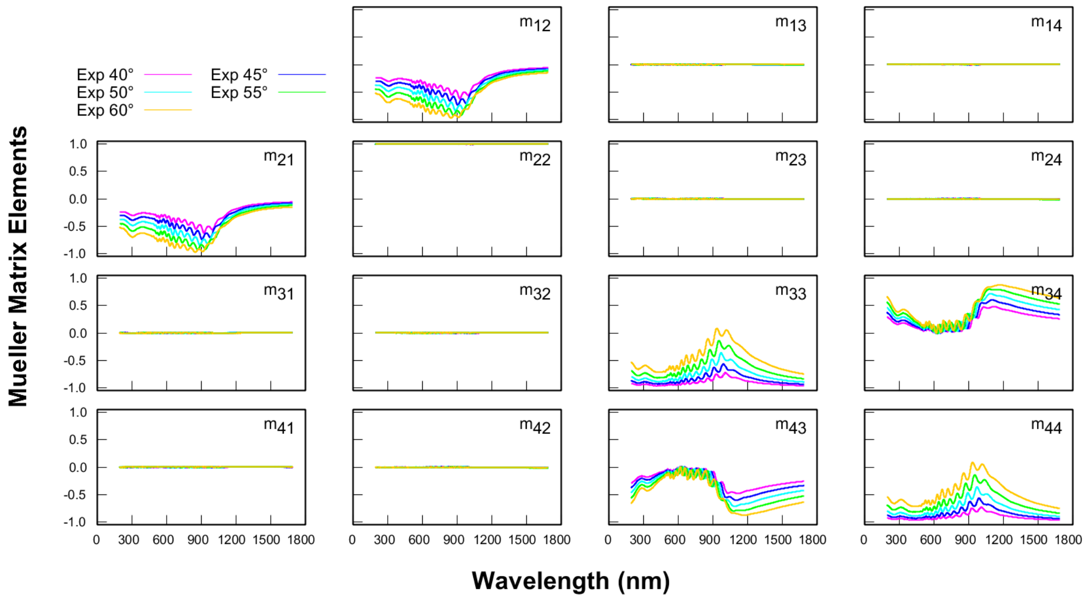

Figure S9: Mueller Matrix elements for a selected TaS_2 flake on a silicon substrate. Note that the zeros in the off-diagonal 2×2 sub-blocks prove the uniaxial nature of this material.

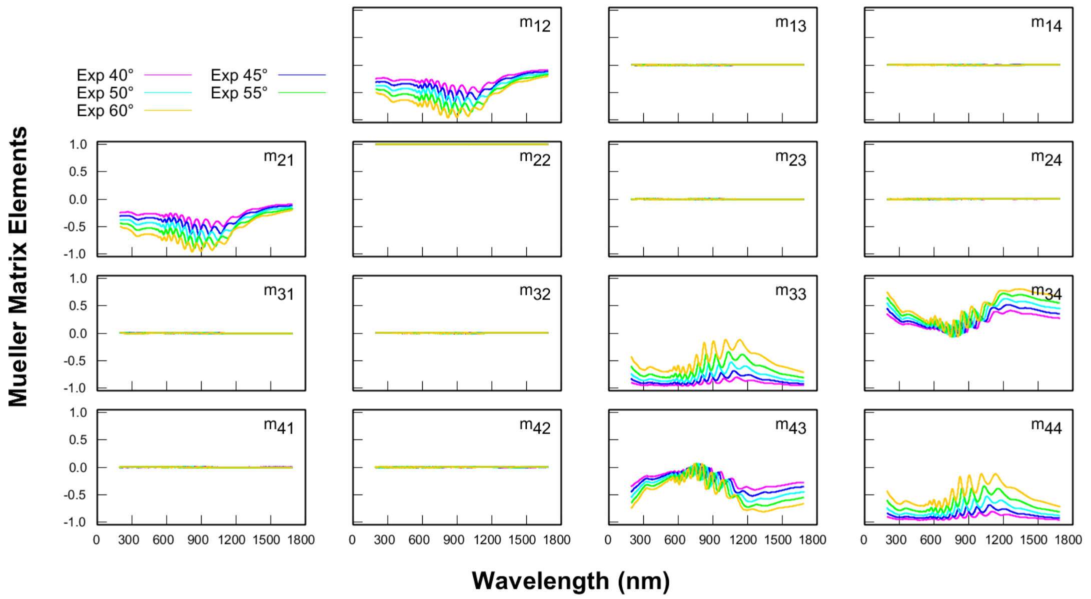

Figure S10: Mueller Matrix elements for a selected $TaSe_2$ flake on a silicon substrate. Note that the zeros in the off-diagonal 2×2 sub-blocks prove the uniaxial nature of this material.

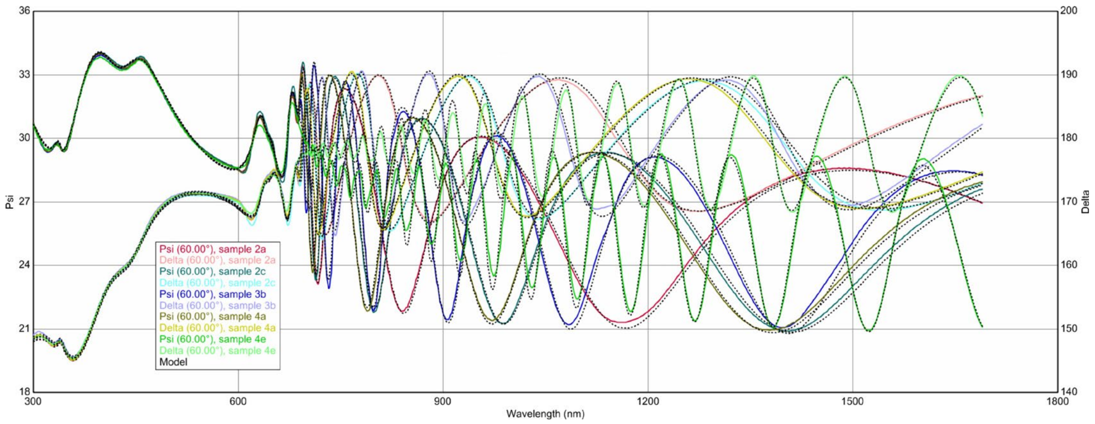

Figure S11: Fidelity of the model fit to measurement data for MoS_2.

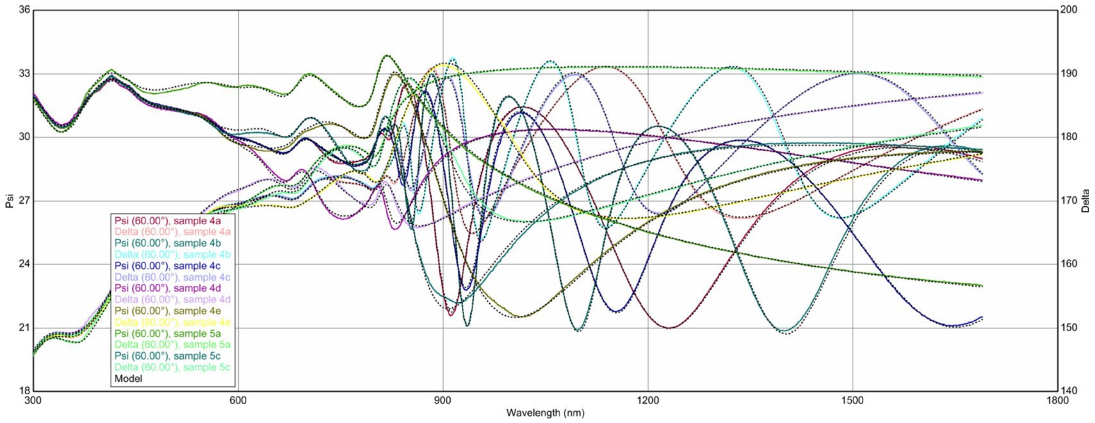

Figure S12: Fidelity of the model fit to measurement data for $MoSe_2$.

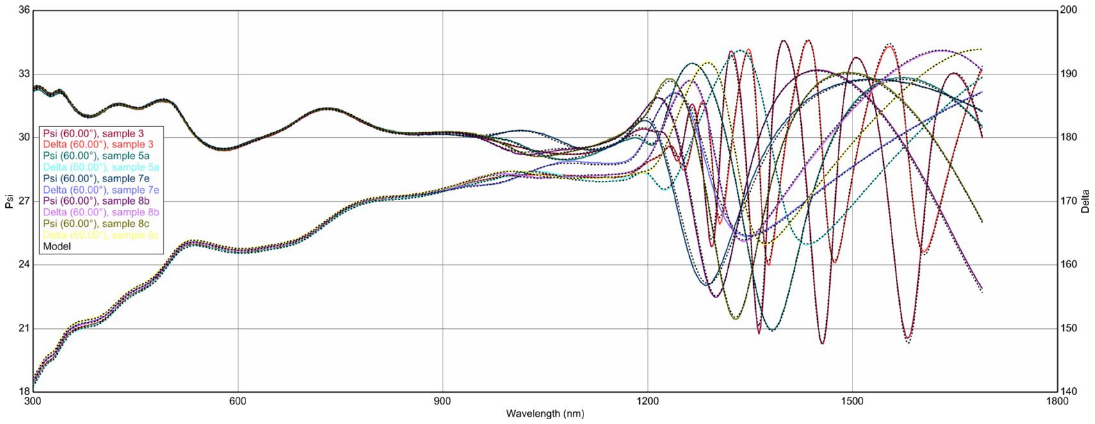

Figure S13: Fidelity of the model fit to measurement data for $MoTe_2$.

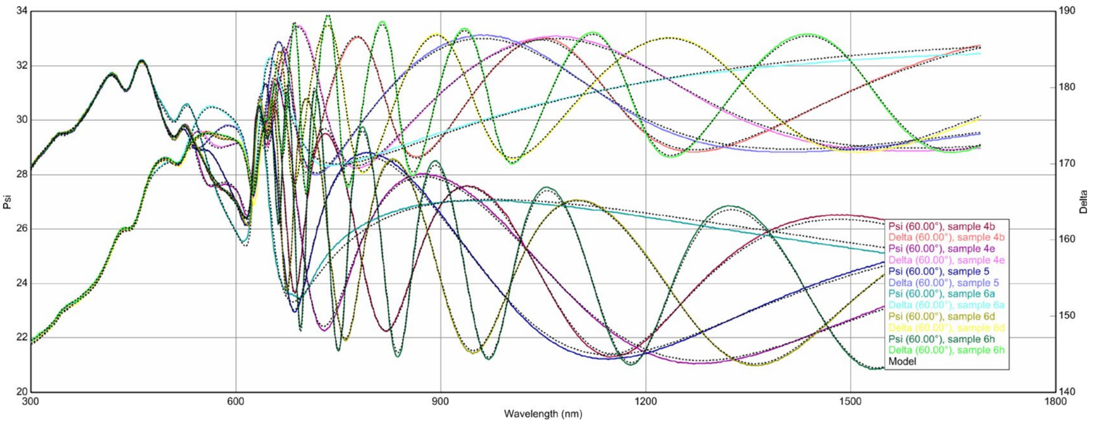

Figure S14: Fidelity of the model fit to measurement data for WS_2.

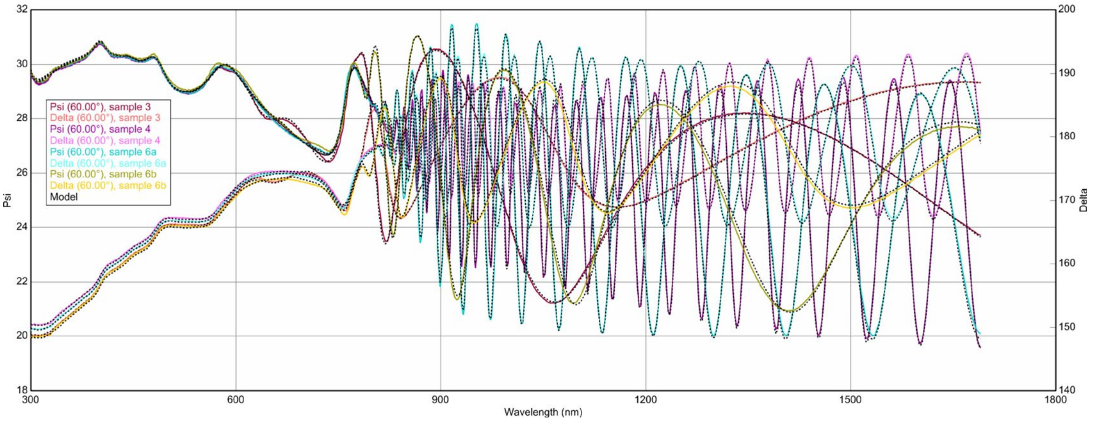

Figure S15: Fidelity of the model fit to measurement data for WSe_2.

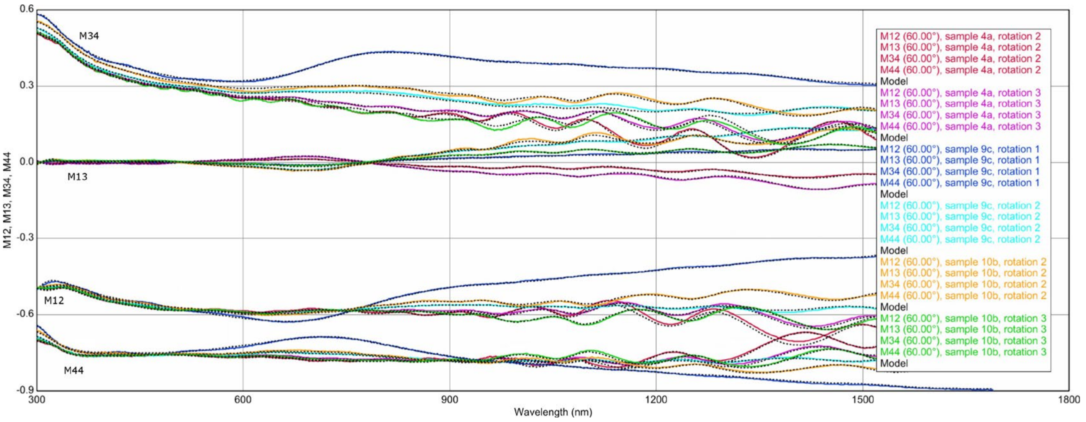

Figure S16: Fidelity of the model fit to measurement data for WTe_2.

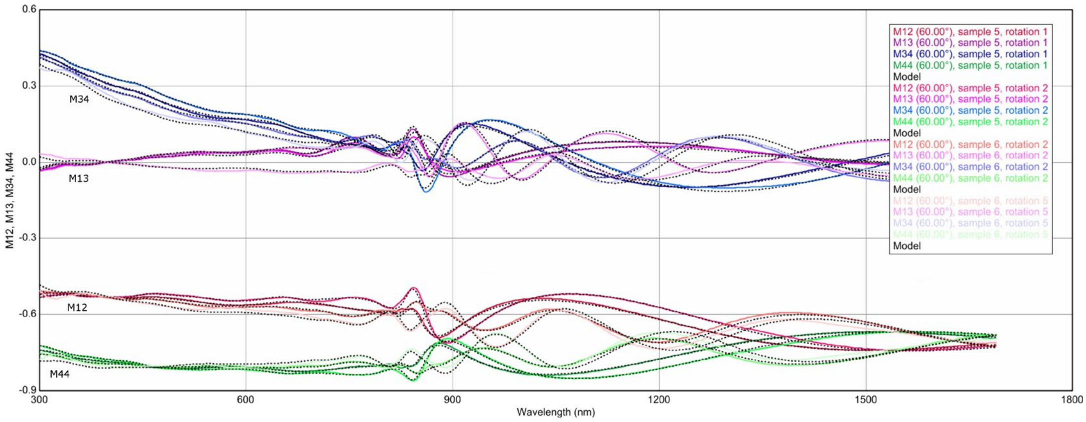

Figure S17: Fidelity of the model fit to measurement data for ReS_2.

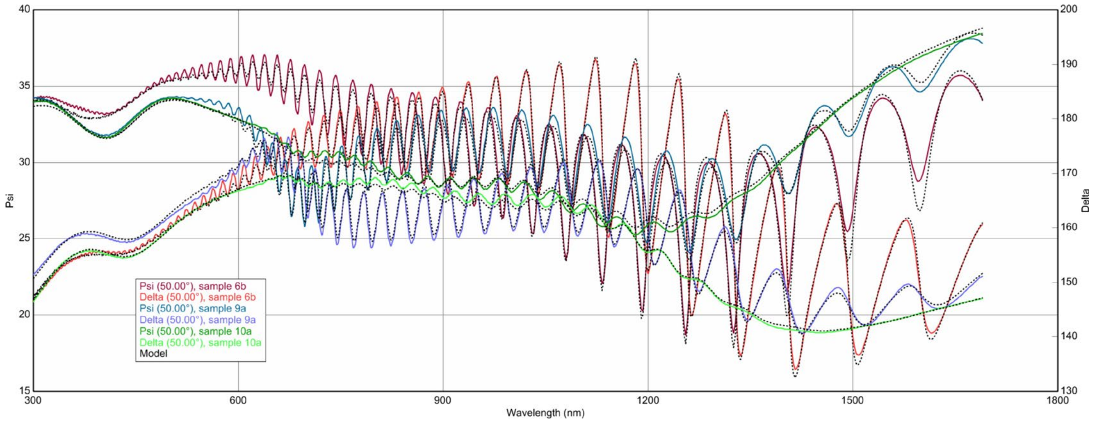

Figure S18: Fidelity of the model fit to measurement data for $NbSe_2$.

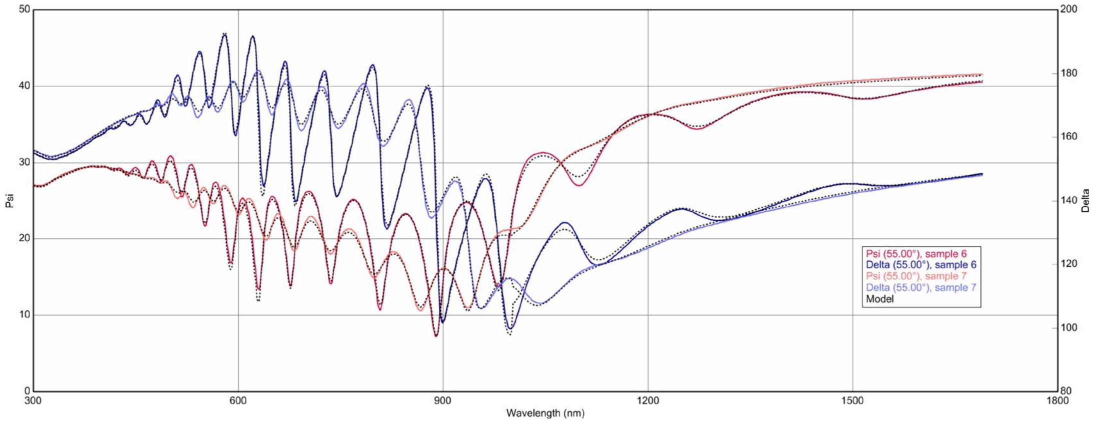

Figure S19: Fidelity of the model fit to measurement data for TaS_2.

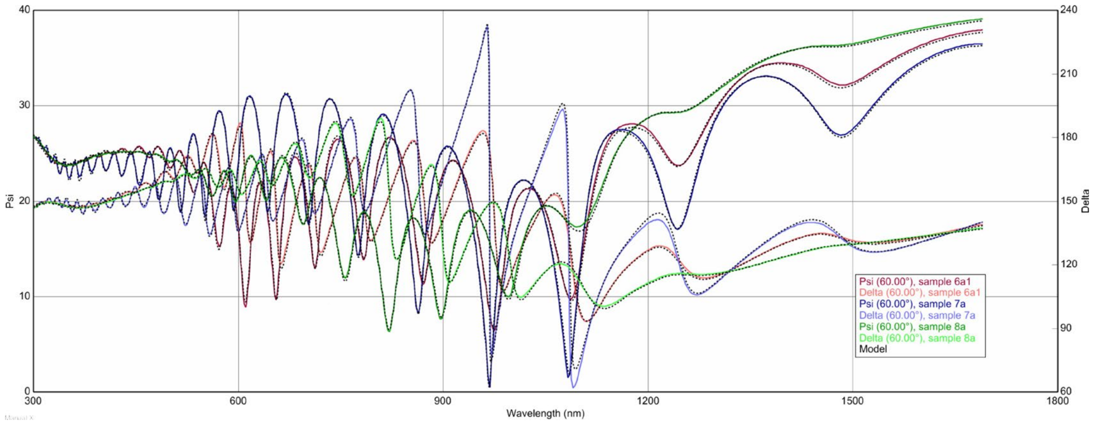

Figure S20: Fidelity of the model fit to measurement data for $TaSe_2$.

Tables of ellipsometric models' parameters

Table S1: Comparison of ellipsometric fitting parameters for uniaxial TMDs. The fitted thickness values agree very well with (where available) reference thickness measurements obtained with a profilometer.

		Thickness			
Material	Sample	reference (nm)	fitted (nm)	nonuniformity (%)	Roughness (nm)
MoS_2	2a	301	297	0	1.5
	2c	364	366	0	1.5
	3b	600	546	1.5	1.2
	4a	359	359	0	1.3
	4e	1830	1778	3.4	1.2
$MoSe_2$	1	108	113	0	1.2
	2	123	124	0	0.8
	4a	324	301	0	1.2
	4b	538	524	0	1.1
	4c	418	418	7.6	1.1
	4d	75	76	22.4	0.8
	4e	118	118	0	1.3
	5a	24	30	20.2	0.9
	5b	75	77	23.6	2.0
	5c	93	100	0	1.1
$MoTe_2$	3	1600	1535	1.5	1.4
	5a	428	430	0	1.5
	7e	259	253	9.2	1.2
	8b	400	388	3.6	1.1
	8c	440	403	0.7	0.9
WS_2	3a	1140	1165	0	1.2
	3b	454	456	0.1	1.4
	3d	1356	1356	0.1	1.3
	4b	328	325	0	1.4
	4c	2325	2247	0	1.3
	4e	183	184	0	1.5
	5	164	163	0.1	1.5
	6a	91	81	0.1	1.5
	6d	369	389	0	1.5
	6h	660	664	0	1.4
WSe_2	3	300	273	4.5	1.5
	4	3601	3829	0.3	0.8
	6a	–	2033	0.9	1.1
	6b	613	562	3.2	1.7
$NbSe_2$	6b	52	52	0.4	6.1
	9a	122	122	0	3.5
	10a	373	372	0	7.4
TaS_2	6	120	120	0.5	2.7
	7	250	269	0	2.2
$TaSe_2$	6a1	102	102	0	3.3
	7a	74	70	0.7	3.8
	8a	176	173	0.2	3.6

Table S2: Comparison of ellipsometric fitting parameters for bianisotropic TMDs. The fitted thickness values agree very well with (where available) reference thickness measurements obtained with a profilometer, as do the relative rotation angles. †Note, that sample 7 of ReS_2 is L-shaped and the two arms have different thicknesses as confirmed by profilometer measurements and ellipsometry fitting.

		Thickness			Rough-	Orientation angle	
Material	Sample	reference (nm)	fitted (nm)	nonuniformity (%)	ness (nm)	reference (degrees)	fitted (degrees)
ReS_2	5	196	203	1.2	1.5	-1.6	35.8
			208	2.7	1.6	-16.2	21.8
	6	483	483	2.5	1.6	15.3	46.4
			512	2.9	1.8	81.2	110.4
	7†	191	190	0.5	1.4	-22.3	-7.7
		681	645	5.9	1.8	42.3	57.7
WTe_2	4a	256	233	0.9	0.2	19.6	10.7
			251	3.4	0.2	31.1	22.0
			235	1.9	0.4	46.0	37.9
	5	410	403	1.3	0.3	-1.5	-86.3
			417	1.6	0.5	41.5	-41.7
			460	1.6	0.4	62.8	-20.2
	9c	440	397	1.1	0.4	0.8	-80.4
			412	1.9	0.3	46.3	-35.0
			372	6.6	0.3	86.6	6.1
	10b	233	279	0.4	0.7	45.1	-40.4
			272	1.5	0.2	73.0	-15.3

Supporting Notes

Supporting Note S1: Uncertainty of optical constants.

Evaluation of inaccuracies of extracting the optical parameters from ellipsometric measurements is a very challenging task and cannot be done unequivocally, since the technique is not a direct method. The uncertainty can be influenced by systematic errors of the measurement itself like beam divergence, angle of incidence, sample position, etc, however, the biggest impact on the trustworthiness of the extracted data stems from the optical model. Its validity can be judged by the fit quality and statistically by the Mean Squared Error (MSE), which is a prime parameter used for evaluating the model and how it fits the measured data. However, the MSE parameter alone is merely an indicator that in the case of yielding small values the model might be true. More importantly, it does not take into account what is the sensitivity of the model to the various fitting parameters.

Thus, another way to evaluate the model is the uniqueness test. In this test of the model a given parameter, for example the layer thickness, is fixed (in a given range) at various values while all the other ones are fitted. The width (or more genrally the shape) of the minimum of the MSE curve as a function of the chosen parameter shows the uncertainty of the model. However, considering the fact that many parameters of the model influence the final result and their impact may be vary depending on a particular spectral range, using this measure alone for the evaluation of uncertainties of optical constants may be insufficient as well.

A more systematic way of evaluating the goodness of the proposed models and their fits is done using a Fit Parameter Error Estimation tool that is included in the analysis software (CompleteEASE). (i) The first procedure is based on analyzing random errors to show the influence of the measurement uncertainties. It reanalyzes a randomized set of trial experimental

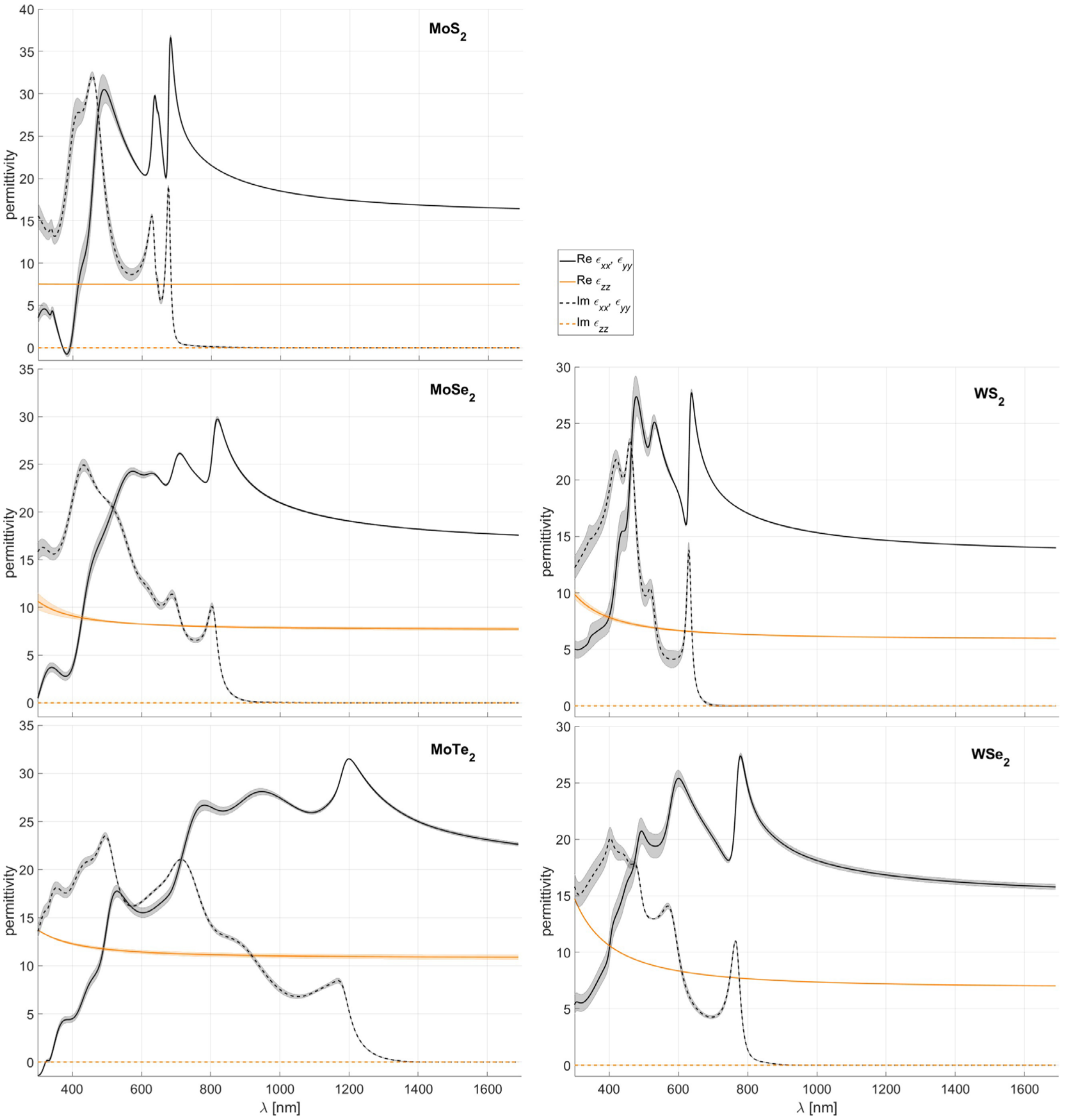

Figure S21: Uncertainty of the complex permittivity for uniaxial semitransparent TMDCs.

data based on the original measurements and refits many sets with the same model. This gives an estimate of the uncertainty for individual fitting parameters as well as the optical constants. (ii) Another set of procedures evaluates the influence of systematic errors coming from such sources as angle offset, wavelength shift, Ψ, and Δ offset or inaccuracies of the complex refractive index of the substrate. (iii) Finally, magnitudes of the fitting errors are tested by adding or subtracting the same magnitude of error at every wavelength to the existing data set and refitting the manipulated data.

Our analysis shows that both random and systematic errors (tested as described above) give a minor contribution to the overall uncertainty of the dielectric functions. On the other hand, the magnitude of the fit error shows the largest influence on the models' spectral sensitivity comes from the distinct optical properties of the subsequent materials. For uniaxial semitransparent TMDs the permittivity errors shown in Figure S21 are negligible in the transparent regions and increase for shorter wavelengths in the absorption bands. This is a result of a lower sensitivity of ellipsometry (the method) itself due to high absorbance in the materials and an influence of the surface roughness, which plays an important role in determining the optical properties of the materials, especially in the UV region.

In the case of bi-anisotropic materials (see Figure S22), ReS_2 shows similar properties in terms of uncertainties of optical parameters to the above examples, as it is also transparent at long wavelengths. In contrast, WTe_2 exhibits comparatively larger errorbars with an increase of the wavelength for the in-plane components and a pronounced uncertainty for the out-of-plane

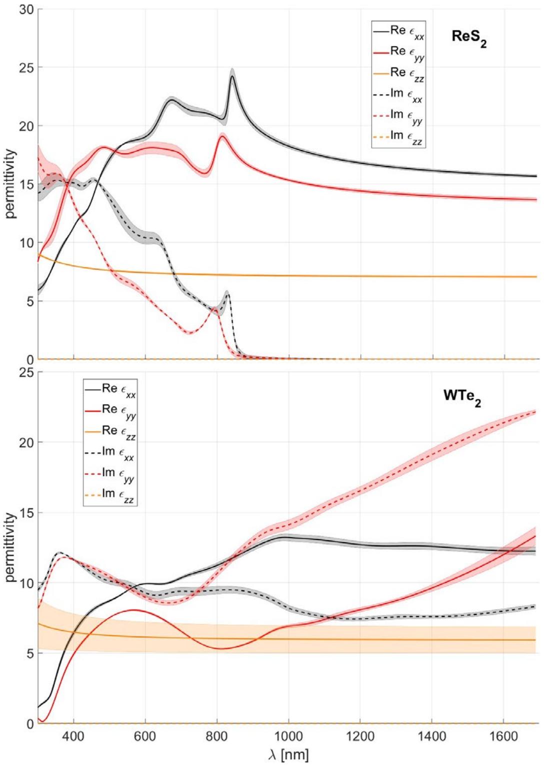

Figure S22: Uncertainty of the complex permittivity for bi-anisotorpic TMDCs.

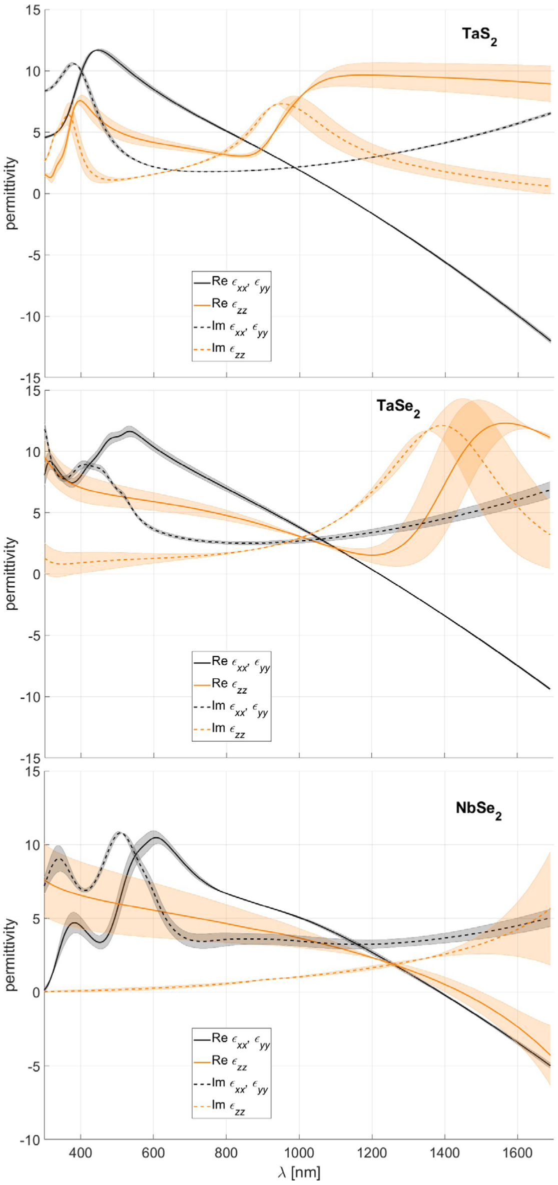

Figure S23: Uncertainty of the complex permittivity for metalic TMDCs.

component in the entire spectral region. The latter one is attributed to the overall large absorptivity of the material in the whole spectral range. This was partially mitigated by preparation of only thin samples, however, due to the mechanical properties of WTe_2 we were only able to obtain flakes with thicknesses down to ∼ 200 nm. Unfortunately, ca. 200 nm WTe_2 flakes still absorb a significant amount of light. Thus, the measured ellipsometric curves lack clear and deep interference features despite a SiO_2 layer between the WTe_2 flakes and the Si substrate. These limitations of preparing WTe_2 flakes led to a low sensitivity of the model to the ε_{zz} component.

Metallic TMDs show enhanced errorbars in the spectral regions with high absorptivity, as illustrated in Figure S23, resulting in a decrease of the interferometic spectral features – see Figure S8–Figure S10 – which leads to high uncertainty of out-of-plane components. It is important to stress that this analysis does not evaluate the overall uncertainty of the ellipsometric technique but rather shows the sensitivity of the models resulting from unique material properties of subsequent samples
